# Tissue signals imprint Aiolos expression in ILC2s to modulate type 2 immunity

**DOI:** 10.1038/s41385-021-00431-5

**Published:** 2021-08-04

**Authors:** Jinxin Qiu, Jingjing Zhang, Yan Ji, Hanxiao Sun, Zhitao Gu, Qiangling Sun, Meizhu Bai, Jue Gong, Jupei Tang, Yunpeng Zhang, Shiyang Li, Zhen Shao, Jinsong Li, Huiming Sheng, Lei Shen, Ju Qiu

**Affiliations:** 1 CAS Key Laboratory of Tissue Microenvironment and Tumor, Shanghai Institute of Nutrition and Health, University of Chinese Academy of Sciences, Chinese Academy of Sciences, Shanghai, China; 2 Shanghai Institute of Immunology, Shanghai Key Laboratory for Tumor Microenvironment and Inflammation, Shanghai Jiao Tong University School of Medicine, Shanghai, China; 3 Department of Laboratory Medicine, Tongren Hospital, Shanghai Jiao Tong University School of Medicine, Shanghai, China; 4 Department of Thoracic Surgery, Shanghai Chest Hospital, Shanghai Jiao Tong University, Shanghai, China; 5 Thoracic Cancer institute, Shanghai Chest Hospital, Shanghai Jiao Tong University, Shanghai, China; 6 State Key Laboratory of Cell Biology, Shanghai Key Laboratory of Molecular Andrology, Shanghai Institute of Biochemistry and Cell Biology, Center for Excellence in Molecular Cell Science, Chinese Academy of Sciences, Shanghai, China; 7 Department of General Surgery, Tongren Hospital, Shanghai Jiao Tong University School of Medicine, Shanghai, China; 8 Advanced Medical Research Institute, Shandong University, Jinan, Shandong China; 9 CAS Key Laboratory of Computational Biology, CAS-MPG Partner Institute for Computational Biology, Shanghai Institute of Nutrition and Health, Chinese Academy of Sciences, Shanghai, China

## Abstract

Group 2 innate lymphoid cells (ILC2s) manifest tissue heterogeneity and are crucial modulators of regional immune responses. The molecular mechanisms regulating tissue ILC2 properties remain elusive. Here, we interrogate the signatures of ILC2s from five tissues at the transcriptome and epigenetic level. We have found that tissue microenvironment strongly shapes ILC2 identities. The intestine induces Aiolos^+^ILC2s, whereas lung and pancreas enhance Galectin-1^+^ILC2s. Though being a faithful gut ILC2 feature under the steady state, Aiolos is induced in non-intestinal ILC2s by pro-inflammatory cytokines. Specifically, IL-33 stimulates Aiolos expression in both human and mouse non-intestinal ILC2s. Functionally, Aiolos facilitates eosinophil recruitment by supporting IL-5 production and proliferation of ST2^+^ILC2s through inhibiting PD-1. At the epigenetic level, ILC2 tissue characters are imprinted by open chromatin regions (OCRs) at non-promoters. Intestinal-specific transcription factor aryl hydrocarbon receptor (Ahr) binds to *Ikzf3* (encoding Aiolos) locus, increases the accessibility of an intestinal ILC2-specific OCR, and promotes the *Ikzf3* transcription by enhancing H3K27ac. Consequently, Ahr prevents ILC2s entering an “exhausted-like” state through sustaining Aiolos expression. Our work elucidates mechanism of ILC2 tissue adaptation and highlights Aiolos as a potential target of type 2 inflammation.

## Introduction

Group 2 innate lymphoid cells (ILC2s) are a subset of ILCs that lack T and B cell antigen-specific receptors.^1–4^ ILC2s mirror the CD4^+^ type 2 T helper cells in terms of master transcription factor GATA3 expression and functional cytokine production.^1,5,6^ Both ILC2s and Th2 cells play fundamental roles in expulsion of parasites and induction of allergy through secretion of IL-5 and IL-13, which mediate multiple effects including recruitment of myeloid cells, eosinophil maturation, smooth muscle contraction, and mucin production from epithelial cells.^7^ Importantly, ILC2s are found to be more dominant source for type 2 cytokines than Th2 cells in tissues under the steady state, and are key drivers for allergic responses at early stage of diseases.^8–10^ ILC2s have also been reported to promote epithelial regeneration by producing amphiregulin (AREG).^11^ In addition, ILC2s have recently been indicated to have anti-tumor capacity.^12–15^ Therefore, ILC2s serve as promising therapeutic target not only for infectious diseases or allergy, but also for tissue damage and tumor.

Developed from the bone marrow (BM) common helper-like innate lymphocyte progenitors (ChILP, lineage^−^CD127^+^Id2^+^α_4_β_7_^+^),^16^ ILC2s accumulate at mucosal areas starting at an early age and populate locally through homeostatic expansion.^17,18^ As a result, tissue-resident ILC2s constantly crosstalk with environmental cues and are imprinted with specific characters that may endow ILC2s with site-specific functions.

One reflection of the tissue ILC2 heterogeneity is the preference of cytokine receptor expression.^19^ IL-18R has been shown to be highly expressed by skin ILC2s.^20^ Small intestine (SI) is abundant for ILC2s expressing IL-17RB, the receptor for IL-25.^21^ The IL-17RB^+^ILC2s have also been found in the lung upon injection of IL-25 and are defined as inflammatory ILC2s (iILC2s).^22^ ST2, the receptor for IL-33, is expressed by ILC2s distributed broadly in many organs including BM ILC2 progenitors.^20,23^ Correspondingly, IL-33 has been implicated with diseases from multiple tissues, including the lung, pancreas, intestine, and fat.^11,24–28^ IL-33 facilitates egress of ILC2s from BM, and is essential for the maintenance and cytokine production of ILC2s during inflammation, as well as generation of ILC2 memory.^24–26^ IL-33 efficiently expands ILC2s and results in systemic type 2 responses, and is indicated to be applicable in ILC2-related therapy for treatment of diseases, such as graft-versus-host disease or specific types of tumors.^13,29–31^ Therefore, the elaboration of ST2^+^ILC2 properties in different microenvironments may facilitate targeting ILC2s in a tissue-specific manner.

ILC2 tissue specificity has been previously investigated by bulk cell RNA-seq, single-cell RNA-seq, and mass cytometry.^20,32,33^ However, transcriptome profiles of ILC2s from the large intestine (LI) and pancreas, two organs in which ILC2s have recently been shown to be present in cancer patients and to be relevant to anti-tumor functions of ILC2s, remain unknown.^13,14,33^ In addition, it is not clear if the signatures of ILC2s from one specific tissue (donor tissue) will change as the cells are relocated to another tissue (host tissue).

In parallel with the transcriptome profiles, the heterogeneity of immune cells is also imprinted at the epigenetic level. Open chromatin regions (OCRs) at proximal and distal enhancers have been shown to play a fundamental role in lineage commitment of immune cells.^34,35^ Integrated analysis of assay for transposase-accessible chromatin with sequencing (ATAC-seq) and chromatin immunoprecipitation sequencing (ChIP-seq) reveals that transcription factors play essential roles in regulating chromatin accessibilities, histone modifications, and gene expression.^36,37^ Transcription factors binding to OCRs in multiple tissue ILC2s, which has not been explored before, may be critical upstream events in determining ILC2 tissue properties.

In this study, we interrogated the transcriptomic and epigenetic features of ST2^+^ILC2s from the BM, pancreas, lung, and LI, as well as the features of KLRG1^+^ILC2s from SI. We identified Galectin-1 as a lung/pancreas ILC2 feature and Aiolos as an intestinal ILC2 signature under the steady state. Aiolos belongs to the Ikaros zinc-finger family transcription factors and is expressed by various types of immune cells.^38^ Through forming homodimer or forming heterodimer with Ikaros, or interaction with other non-Ikaros zinc finger family transcription factors and epigenetic modulators, Aiolos regulates gene expression by directly binding to target DNA with its C2H2 Krüppel-like zinc-finger motifs.^38^ Aiolos has been shown to regulate the function of ILC subsets. For example, Aiolos is important for the maturation of splenic CD11b^high^CD27^–^NK cells and is required for the anti-viral capacity of NK cells through cell-intrinsically sustaining IFN-γ and granzyme B expression by NK cells.^39^ Nevertheless, mice with defective Aiolos expression in NK cells have an enhanced anti-tumor response in vivo.^39^ Recent research studies have found that Aiolos is expressed by a transitional ILC3-ILC1 subset, cooperates with T-bet to extinguish the ILC3 program, and facilitates the conversion of ILC3 to ILC1, a process possibly involved in the pathogenesis of inflammatory bowel disease.^40–42^ So far, studies on the role of Aiolos in ILC2s have been lacking. In this research, we investigated the molecular mechanisms regulating Aiolos expression and the function of Aiolos in tissue ILC2s. Our research is valuable for understanding the molecular regulation of ILC2 tissue specificity and provides potential insights for specifically targeting ILC2s during diseases.

## Results

### Tissue ILC2s share sub-clusters with similar characteristics

To interrogate the heterogeneity of mouse tissue ILC2s, we performed scRNA-seq on ILC2s sorted from five different organs, including BM, LI, lung, pancreas, and SI using flow cytometry. Lin^–^ST2^+^CD25^+^ was used as an identity for sorting ST2^+^ILC2s from the BM, LI, lung, and pancreas, more than 95% of which represents genuine ILC2s (Lin^–^CD45^+^CD127^+^GATA3^high^ cells) (Supplementary Fig. S1a, c, e). Since SI ILC2s express low level of ST2,^20^ Lin^–^CD45^+^Thy1^low^KLRG1^high^ was used as markers for sorting SI ILC2s, more than 97% of which match identities of gold standard of ILC2s (Lin^–^CD45^+^CD127^+^GATA3^high^ cells) (Supplementary Fig. S1b, d, e). T-distributed stochastic neighbor embedding (t-SNE) analysis identified six to eight sub-clusters from ILC2s of each tissue (Supplementary Fig. S2a). We observed that ILC2s from the same tissue manifested relatively low heterogeneity (Supplementary Fig. S2a). But a few sub-clusters appeared to be more separate from others, such as cluster 7 from the BM ILC2s, cluster 6 from the LI ILC2s, and cluster 5 and 6 from the pancreas ILC2s (Supplementary Fig. S2a). Genes highly expressed by one sub-cluster compared to each of the other sub-cluster in a 1 versus 1 manner were specifically noticed. We found that sub-clusters with featured expression of *Lgals1* together with several other characteristics were shared among all five tissue ILC2s (Supplementary Fig. S2b). The *Lgals1*^high^ clusters in BM, lung, and pancreas ILC2s had higher level of *S100a4* and *S100a6*, whereas enriched *Tnfsf8* expression in *Lgals1*^high^ clusters was found in all the tissue ILC2s except for BM and SI ILC2s (Supplementary Fig. S2b). Interestingly, *Lgals1*^high^ clusters in intestinal ILC2s showed distinguished expression of *Il17a* (Supplementary Fig. S2b). BM, lung, and pancreas ILC2s were found to contain a sub-cluster with featured expression of *Notch2* and genes encoding epigenetic modulators, including *Kdm6b*, *Kmt2d*, and *Cbx3* (Supplementary Fig. S2c). This possibly indicates that Notch signaling supporting the development and expansion of ILC2s is coupled with epigenetic activities.^43^

Strikingly, cluster 1 from LI ILC2s and cluster 3 from SI ILC2s manifested high expression of *Il5* and *Il13*, indicating them being an activated subpopulation (Supplementary Fig. S2d).^44^ *Tnfrsf9*, *Cxcr6*, and *Dgat1* were identified together as featured genes of this activated ILC2 cluster (Supplementary Fig. S2d). We speculated that common signatures in specific clusters could predict co-expression in a cell subpopulation. Using the IL-5-RFP (Red5) mouse,^45^ we verified the enriched expression of *Il13* mRNA in IL-5-RFP^+^ILC2s from SI, and concomitant abundance of *Il5*, *Il13*, and *Tnfrsf9* mRNA in IL-5-RFP^+^ILC2s from LI (Supplementary Fig. S2e). At the protein level, surface expression of 4-1BB (encoded by *Tnfrsf9*) on ILC2s was hardly detected under the steady state (Supplementary Fig. S2f). However, phorbol 12-myristate 13-acetate (PMA) and ionomycin stimulation significantly boosted both surface and intracellular 4-1BB, which was specifically highly expressed by IL-5^+^ILC2s rather than IL-5^–^ILC2s in the LI (Supplementary Fig. S2f, g). The data indicate that 4-1BB mark an activated ILC2 subpopulation and that signatures of sub-clusters identified by single-cell sequencing predict their co-expression as markers for tissue ILC2 sub-populations.

### ILC2s have distinct tissue properties

**Fig. 1:**
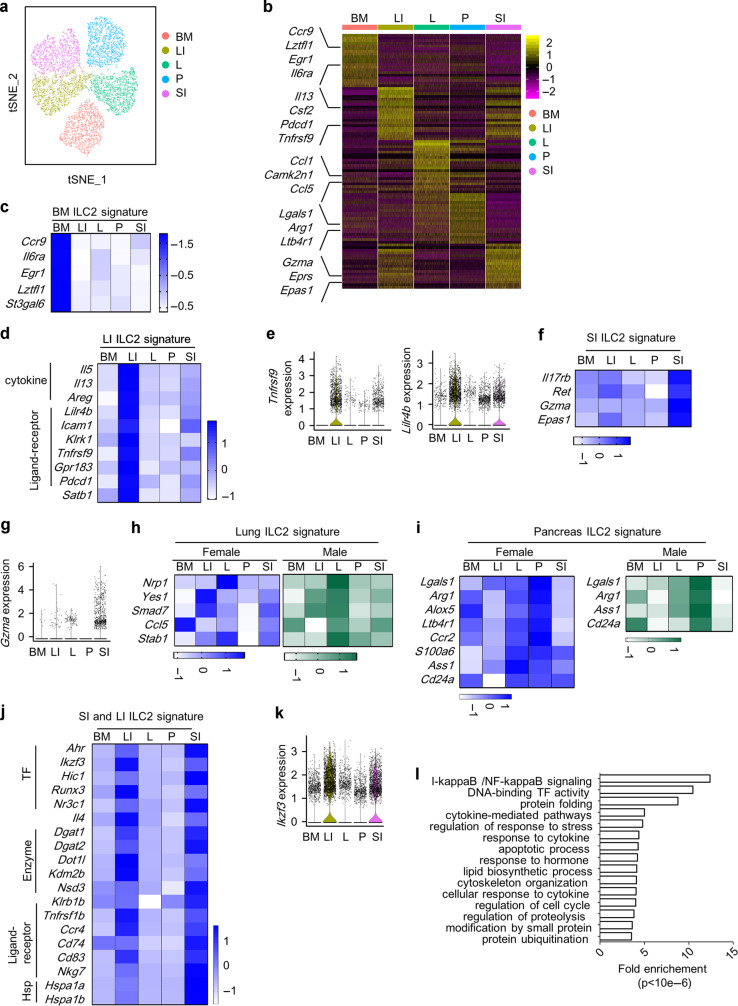
Tissue ILC2s have distinct signatures. **a**, **b**, **e**, **g**, **k**, **l** Analysis of aggregated and downsampled single-cell data from tissue ILC2s was performed using Seurat. **a** t-SNE plot shows the distinct clusters of tissue ILC2s. **b** Heatmap shows the top 30 differentially expressed genes from tissue ILC2s performed using “1 versus all” comparison. **e**, **g**, **k** Violin plots show differentially expressed genes by tissue ILC2s. **c**, **d**, **f**, **h**, **i**, **j** Expression of ILC2 tissue-specific genes was verified in purified ILC2s from female mice (**c**, **d**, **f**, and **j**) or both genders (**h** and **i**) by real-time RT-PCR. **c**, **d**, **f**, **h**, **i**, **j** Heatmaps show relative fold of gene expression of representative tissue signatures relative to the expression by BM ILC2s. Black blocks indicate the gene was not detected in the tissue ILC2s by real-time RT-PCR. Scale bar indicates *z*-score of gene expression relative to *Actb*. **l** Gene ontology analysis was performed on LI and SI ILC2 common signature genes. BM bone marrow, LI large intestine, SI small intestine, P pancreas, L lung.

Despite sub-clusters with similar features were found in ILC2s from different tissues, analysis aggregating downsampled ILC2s from each tissue revealed strong heterogeneity (Fig. 1a, b and Supplementary Data 1). We performed pairwise comparison on single-cell data between tissue ILC2s, and genes commonly upregulated in one tissue ILC2s compared to all other four tissue ILC2s were designated as “ILC2 tissue-specific genes” (ILC2 TS genes) (Supplementary Data 1). To compensate for the potential gender-biased results obtained from scRNA-seq data of male ILC2s, the expression of representative ILC2 TS genes was evaluated using female tissue ILC2s by quantitative PCR. Taking previously reported ILC2 tissue signatures as controls including *Ccr9* (for BM ILC2), *Il5* (for LI ILC2), and *Il17rb* (for SI ILC2), we identified and confirmed several tissue features for ILC2s, including *Il6ra* and *Lztfl1* for BM ILC2s, *Tnfrsf9* and *Lilr4b* for LI ILC2s, *Ret*, *Gzma*, and *Epas1* for SI ILC2s, *Nrp1* for lung ILC2s, and *Lgals1*, *Alox5*, and *Arg1* for pancreas ILC2s (Fig. 1c–i). Intriguingly, peaked expression of *Yes1*, *Smad7*, *Ccl5*, and *Stab1* in lung ILC2s, and peaked expression of *Ass1* and *Cd24a* in pancreas ILC2s were only found in male but not female individuals (Fig. 1h, i), suggesting that sex hormones affect the establishment of lung and pancreas ILC2 tissue features.^46^ Notably, *Lgals1* was also expressed at a higher level in lung ILC2s compared with BM and intestinal ILC2s especially in male mice (Fig. 1i).

Hierarchical clustering analysis indicated a closer relationship of LI and SI ILC2s (Supplementary Fig. S3a, b). We then confirmed the differential expression of genes, which were concomitantly higher in LI and SI ILC2s compared to ILC2s from each of the other three tissues, from female mice using quantitative PCR (Fig. 1j and Supplementary Data 1). A series of genes were verified to be intestinal ILC2 features, including aryl hydrocarbon receptor (Ahr) that has been reported to be an intestinal ILC2 character,^47^ and *Ikzf3* encoding the transcription factor Aiolos (Fig. 1j, k). Gene ontology analysis showed that intestinal ILC2s signature genes were significantly enriched in pathways including transcription factor activities, lipid synthesis, cell cycle, cell apoptosis, cytoskeleton organization, protein folding and post-translational modification, and cytokine responses (Fig. 1l), which were not found to be over-presented in non-intestinal ILC2 signature genes (data not shown). This suggests that intestinal ILC2s undertake more active biological processes compared to other tissue ILC2s, and intestinal ILC2s are likely to be more intricately regulated at the molecular level to accommodate their complicated functions.

### Tissue microenvironment actively shapes ILC2 characters

**Fig. 2:**
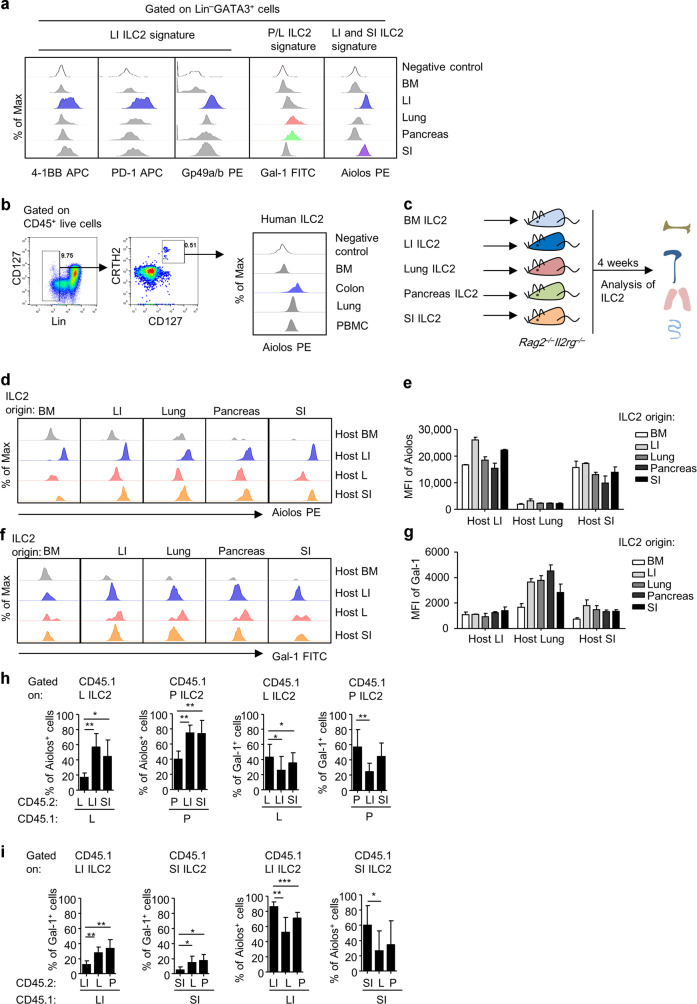
Tissue environment strongly shapes ILC2 identities. **a** The expression of representative ILC2 tissue signatures gated on ILC2s was analyzed by flow cytometry. Intracellular 4-1BB was analyzed when cells were stimulated with PMA and ionomycin. Myeloid cell gate based on FSC and SSC was used as negative control. **b** Histogram shows Aiolos expression gated on human ILC2s from different tissues analyzed by flow cytometry. Negative control was Lin^+^ cells from bone marrow sample. **c** Schematic strategy of ILC2 transfer. **d**–**g** ILC2s were sorted from different tissues and transferred to *Rag2*^*–/–*^*Il2rg*^*–/–*^ mice. Expression of Aiolos (**d** and **e**) and Gal-1 (**f** and **g**) gated on ILC2s (Lin^*–*^GATA3^+^) from different tissues in *Rag2*^*–/–*^*Il2rg*^*–/–*^ hosts was analyzed by flow cytometry 4 weeks after transfer. Mean fluorescence intensity (MFI) of Aiolos and Gal-1 is shown. **h**, **i** Mononuclear cells isolated from different tissues of the CD45.1 and CD45.2 mice were mixed at a 1:1 ratio and cultured for 48 h. The expression of Aiolos and Gal-1 gated on ILC2s (CD45.1^+^Lin^*–*^GATA3^+^ cells) of CD45.1 origin was analyzed by flow cytometry. **e**–**i** Error bars are mean + SEM. **a**–**i** Data are representative of two to four independent experiments. BM bone marrow, LI large intestine, SI small intestine, P pancreas, L lung.

We next confirmed the protein expression of ILC2 tissue signatures by flow cytometry (Fig. 2a). In consistency with the mRNA level, 4-1BB, PD-1 (encoded by *Pdcd1*), and Gp49a (encoded by *Lilr4b*) were highly expressed by LI ILC2s (Fig. 2a). Galectin-1 (Gal-1, encoded by *Lgasl1*) specifically marked pancreas and lung ILC2s (Fig. 2a). Notably, Aiolos was confirmed to be a shared feature for LI and SI ILC2s (Fig. 2a). Strikingly, we found that Aiolos was conservatively presented as an intestinal ILC2 signature over BM, lung, and peripheral blood ILC2s in humans (Fig. 2b). To determine if tissue microenvironment affects ILC2 tissue features, we transferred ILC2s isolated from one specific tissue to immune-deficient *Rag2*^*–/–*^*Il2rg*^*–/–*^ hosts, in which ILC2s from different tissues were analyzed 4 weeks later (Fig. 2c–g). We analyzed the expression of Aiolos as a marker highly expressed by ILC2s of intestine origins, and Gal-1 as a marker of ILC2s from pancreas and lung origins. Interestingly, we found that ILC2s from non-intestinal origins dramatically upregulated Aiolos expression upon settling in the gut, whereas intestinal ILC2s downregulated Aiolos expression upon arrival at the lung (Fig. 2d, e). Likewise, Gal-1 expression in ILC2s of the BM and intestine-origin was enhanced when ILC2s were located at the lung, whereas Gal-1 was reduced in ILC2s derived from lung upon homing to the intestine (Fig. 2f, g). As a note, ILC2s from non-BM origin could hardly traffic to the BM and few cells could be harvested from the BM of the host, probably due to lack of BM-homing receptors (Fig. 2d, f). Next, adaption of tissue ILC2s to the environment was tested ex vivo, by co-culture of isolated mononuclear cells from different tissues identified with separate congenic leukocyte markers (Fig. 2h, i). In consistency with observed in vivo, we found that intestinal cells triggered Aiolos expression and suppressed Gal-1 expression in pancreas and lung ILC2s as soon as 48 h of culture (Fig. 2h). Conversely, pancreas and lung cells promoted Gal-1 expression while inhibited Aiolos expression in intestinal ILC2s (Fig. 2i). Together, the data suggest that tissue microenvironment actively skews ILC2 tissue identities.

### Aiolos is induced in non-intestinal ILC2s by pro-inflammatory cytokines

**Fig. 3:**
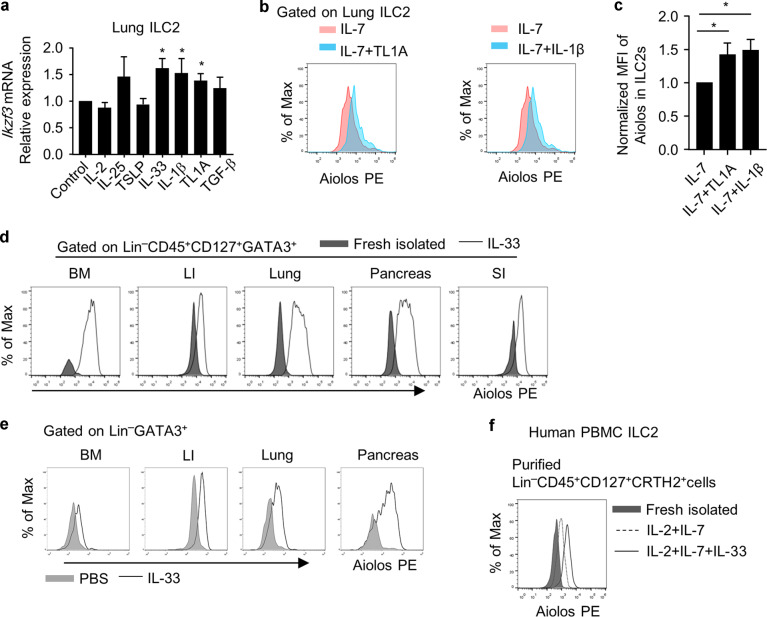
Aiolos is induced in non-intestinal ILC2s by IL-33. **a**–**c** Purified lung ILC2s were treated with indicated cytokines in the presence of IL-7 (10 ng/ml) for 24 h. Concentration for all cytokines were used at 10 ng/ml except for TGF-β (1 ng/ml), TL1A (100 ng/ml) and TSLP (20 ng/ml). **a** mRNA expression of *Ikzf3* was analyzed by Q-PCR. **b** Expression of Aiolos was analyzed by flow cytometry. **c** Mean fluorescence intensity (MFI) of Aiolos is shown. **d** ILC2s from different tissues of wild-type mice were sorted and cultured with IL-7 (10 ng/mL) and IL-33 (10 ng/mL) for 14 days. The expression of Aiolos in IL-33-treated ILC2s compared to freshly isolated ILC2s from indicated organs was analyzed by flow cytometry. **e** Expression of Aiolos in PBS or IL-33 (intraperitoneal injection of IL-33, 500 ng/day/mouse) treated mice gated on ILC2s from indicated organs was analyzed by flow cytometry. Histogram of Aiolos expression is shown. **f** Human peripheral blood ILC2s were isolated and treated with indicated cytokines for 5 days. Histogram shows Aiolos expression analyzed by flow cytometry. **a**, **c** Error bars are mean ± SEM. **d**–**e** BM bone marrow, LI large intestine, SI small intestine, P pancreas, L lung. **a**–**f** Data are representative of two to four independent experiments.

Next, a series of cytokines were used to treat ST2^+^ILC2s in vitro to search for potential factors affecting ILC2 tissue signatures (Fig. 3a and Supplementary Fig. S4a). We found that IL-1β and TL1A promoted both mRNA and protein expression of Aiolos from lung ILC2s in vitro (Fig. 3a–c). Moreover, as a cytokine implicated in systemic allergic responses, IL-33 boosted Aiolos expression in ILC2s from multiple organs both in vitro and in vivo (Fig. 3a, d, e). Importantly, Aiolos expression was also induced in human peripheral blood ILC2s by IL-33, suggesting a conserved regulation of Aiolos by IL-33 (Fig. 3f). Together, the above data indicate that Aiolos could be induced in non-intestine organs by pro-inflammatory triggers such as IL-33, reflecting a plasticity of ILC2 tissue features during diseases.

As a lung/pancreas ILC2 signature, *Lgals1* mRNA expression could be induced in LI ILC2s by IL-2, IL-25 and TGF-β (Supplementary Fig. S4a); however, only TGF-β could induce Gal-1 expression at the protein level (Supplementary Fig. S4b–d). Interestingly, co-presented as sub-cluster marker with *Lgals1* in tissue ILC2s (Supplementary Fig. S2b), *S100a4* and *S100a6* mRNA expression could also be induced by TGF-β (Supplementary Fig. S4e). This implies a role of TGF-β in shaping ILC2 tissue features as well as cluster features.

### Aiolos sustains IL-5 expression in ILC2s by inhibiting PD-1

Aiolos plays a critical role in regulating the function of many types of immune cells, including intestinal ILC3s.^39,40,42,48^ And the induction of Aiolos by IL-33 in ILC2s implies a role of Aiolos in type 2 immunity. We were then prompted to investigate the function of Aiolos in ILC2s. We started the exploration by looking for genes correlated with *Ikzf3* (encoding Aiolos) in mRNA expression at the single-cell level. Twelve genes (including *Rora*, *Gata3*, *Pdcd1*, *Areg*, and *Il5*) positively correlated with *Ikzf3* and three genes (*Hspa1a*, *Jun*, and *Fos*) negatively correlated with *Ikzf3* in expression in LI ILC2s were found (Supplementary Table S1).

**Fig. 4:**
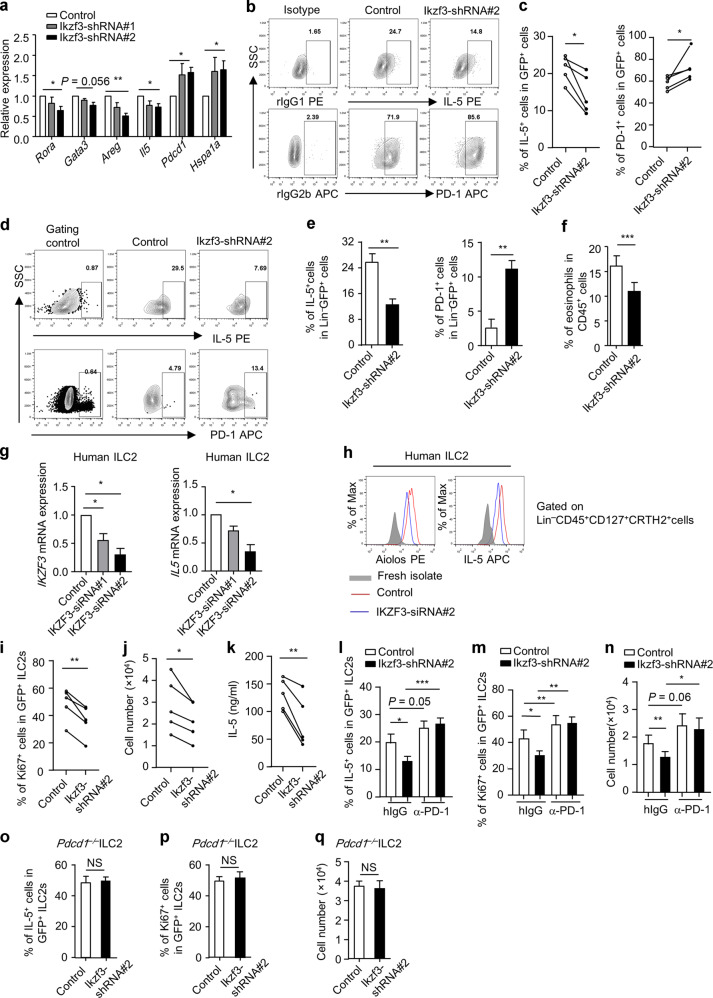
Aiolos supports IL-5 expression by suppressing PD-1. **a**–**c**, **i**–**q** ILC2s were purified from the large intestine of wild-type (**a**–**c**) or *Pdcd1*^*–/–*^ mouse (**o**–**q**) and cultured in vitro in the presence of IL-7 and IL-33 for 5 days. ILC2s were infected with control retrovirus or retrovirus expressing Ikzf3-shRNA#1 or Ikzf3-shRNA#2 (all have GFP as reporter) for two rounds. **a** Expression of indicated genes in purified virus-infected ILC2s (GFP^+^ cells) was analyzed by real-time RT-PCR. **b**, **c**, **i**–**q** GFP^+^ virus-infected cells were purified and cultured starting with equal cell numbers for another 48 h before analysis (in the presence of hamster IgG or α-PD1 antibody for **l**–**n**). Expression of PD-1 (**b** and **c**), IL-5 (**b**, **c**, **l**, **o**), Ki67 (**i**, **m**, **p**) gated on GFP^+^ cells was analyzed by flow cytometry. **b** Isotype control was used to set up gates for IL-5^+^ or PD-1^+^ cells. **j**, **n**, **q** Absolute numbers of ILC2s were shown. **k** Concentration of IL-5 in culture supernatant was analyzed by ELISA. **d**–**f** ILC2s infected with control virus expressing GFP or virus expressing Ikzf3-shRNA#2 were transferred to *Rag2*^*–/–*^*Il2rg*^*–/–*^ mice. **d**, **e** Four weeks after transfer, LI LPLs were isolated and analyzed. Expressions of IL-5 and PD-1 gated on Lin^*–*^GFP^+^ cells from host mice were analyzed by flow cytometry. FACS plots and statistics are shown. **d** Gating control for IL-5 and PD-1 were gated on Lin^+^ cells and Lin^*–*^GFP^*–*^ cells, respectively. **f** Mice were injected with IL-33 for 4 days and were then sacrificed for analysis. Percentages of eosinophils (CD45^+^CD11b^+^Siglec-F^+^) gated on live leukocytes (CD45^+^ cells) are shown. **g**, **h** ILC2s from human PBMC were purified and cultured in the presence with IL-2, IL-7 and IL-33 for 3 days. Cells were treated with control siRNA or siRNA targeting *IKZF3* for 48 h before harvested for analysis. **g** *IKZF3* and *IL5* mRNA expression were analyzed by real-time RT-PCR with *GAPDH* as reference and normalized to the expression of control siRNA group as 1. **h** Brefeldin A was added 2 h before cells were harvested for analysis. Histogram shows expression of Aiolos and IL-5 gated on ILC2s analyzed by flow cytometry. **a**–**q** Data are representative of at least three independent experiments. Error bars are mean + SEM. **c**, **i**, **j**, **k** Connected lines indicate a data pair using cells from same mouse.

To study the function of Aiolos on ILC2s in a cell-intrinsic system, we ablated Aiolos expression in IL-33-expanded LI ILC2s using retrovirus (Supplementary Fig. S5a, b). We found that *Ikzf3* knockdown led to reduced mRNA expression of *Rora*, *Gata3*, *Areg*, and *Il5*, whereas the mRNA expression of *Pdcd1* was enhanced (Fig. 4a). Moreover, expression of *Hspa1a* that negatively correlated with *Ikzf3* in expression was increased upon disturbance of Aiolos (Fig. 4a). At the protein level, GATA3 expression was not altered in *Ikzf3*-knockdown ILC2s (Supplementary Fig. S5c), whereas the decrease of IL-5 accompanied with enhanced PD-1 expression was significant (Fig. 4b, c). In vivo, ILC2s with curtailed Aiolos expression manifested reduced expression of IL-5, AREG and increased PD-1, when transferred to *Rag2*^*–/–*^*Il2rg*^*–/–*^ mice (Fig. 4d, e and Supplementary Fig. S5d, e). And the function of ILC2s in recruiting eosinophils to the intestine was significantly impaired with Aiolos-deficiency (Fig. 4f). Using a CRISPR-associated protein 9 (Cas9)-transgenic mouse, we deleted Aiolos using guide RNAs targeting *Ikzf3* in IL-33-treated ILC2s in vitro (Supplementary Fig. S5f–i). Although Aiolos was only partially deleted in ILC2s probably due to proportional expression of Cas9 (Supplementary Fig. S5f, g and data not shown), reduced IL-5 and increased PD-1 were consistently observed (Supplementary Fig. S5h), whereas GATA3 was similar (Supplementary Fig. S5i). Strikingly, knocking down *IKZF3* in IL-33-treated human ILC2s with siRNA led to significant reduction of Aiolos accompanied with decreased IL-5 at both mRNA and protein levels, suggesting a species-conserved regulation of IL-5 expression in ILC2s by Aiolos (Fig. 4g, h).

PD-1 has been shown to suppress the maintenance and IL-5 production of ILC2s.^49^ Consistently, cell proliferation indicated by Ki67 expression and absolute numbers of ILC2s were reduced upon *Ikzf3* knockdown (Fig. 4i, j), whereas no difference in cell apoptosis was observed (Supplementary Fig. S5j). And a dramatic reduction of IL-5 level in the culture supernatant of *Ikzf3*-knockdown ILC2s was found (Fig. 4k). Strikingly, blockade of PD-1 with a neutralizing antibody reversed the downregulation of IL-5 expression in ILC2s (Fig. 4l), as well as reduced proliferation and numbers of ILC2s (Fig. 4m, n), caused by *Ikzf3* knockdown. In addition, ablation of Aiolos had no effect on IL-5 production (Fig. 4o), cell proliferation, or absolute numbers of *Pdcd1*^*–/–*^ ILC2s (Fig. 4p, q). Together, our data suggest that Aiolos supports the maintenance and IL-5 production by ILC2s through suppressing PD-1.

### Differential OCRs outside of the promoters contribute to ILC2 tissue features

**Fig. 5:**
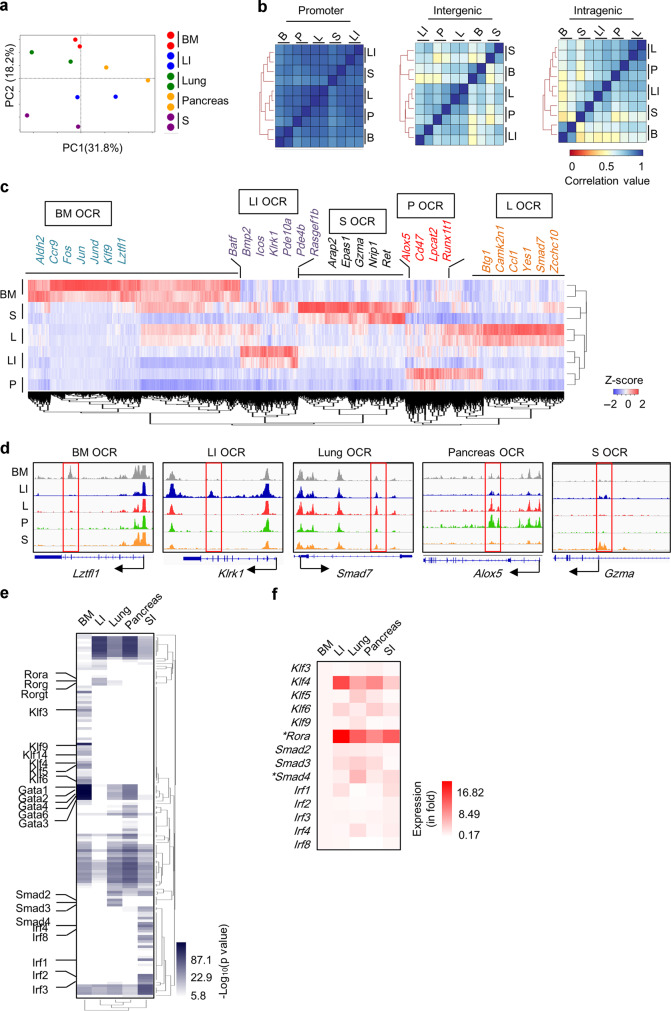
ILC2 tissue-specific open chromatin regions (TS OCRs) correlate with ILC2 tissue signatures. ATAC-seq analysis was performed using ILC2s isolated from different tissues. **a** PCA analysis based on normalized coverage of all combined peaks observed in tissue ILC2s. **b** Correlation analysis was performed on normalized coverage of ILC2 OCRs from indicated regions. Spearman correlation coefficients are shown. **c** Hierarchical clustering and heatmap was generated based on *z*-score of the normalized coverage of ILC2 TS OCRs. Representative ILC2 TS OCRs correlated ILC2 TS genes are shown. **d** Genome browser tracks with red boxes indicating representative ILC2 TS OCRs are shown. **e** Heatmap shows hierarchical clustering based on *p* value ILC2 TS OCRs-enriched motifs. White boxes indicate the motif is not found to be enriched in indicated ILC2 TS OCRs, or the *p* value was higher than the cutoff value (1 × 10^−6^). **f** mRNA expression of indicated genes from isolated tissue ILC2s was analyzed by real-time RT-PCR. Heatmap shows relative average expression of indicated genes normalized to the expression of BM ILC2s as 1. *indicates significant difference in increased expression of tissue-specific genes compared with each of the other tissue ILC2s analyzed from four biological samples.

Our above data indicate that tissue ILC2s are endowed with distinct molecular features by the environment. We then determined to search for cis-regulatory elements imprinting tissue identities of ILC2s by examining the chromatin accessibility of tissue ILC2s by ATAC-seq. From ILC2s of five tissues altogether, we identified 50,039 peaks, majority of which were distributed in promoters, introns, and intergenic regions (Supplementary Fig. S6a, b). And 19,538 peaks were consistently identified to be shared among five tissue ILC2s (Supplementary Fig. S6a). Principle component analysis manifested heterogeneity of OCRs among tissue ILC2s (Fig. 5a). This heterogeneity is mainly contributed by non-promoter regions indicated by correlation analysis on the accessibility of OCRs (Fig. 5b). Through pairwise comparisons looking for statistically differential peaks, we identified ILC2 tissue-specific open chromatin regions (TS OCRs) that were concomitantly higher in ILC2s from one specific tissue than in ILC2s from every other tissue (Fig. 5c). These ILC2 TS OCRs were expected to contain cis-regulatory elements determining the expression of tissue-specific genes. A total of 579–2126 ILC2 TS OCRs were identified from ILC2s of each tissue. Consistently, ILC2 TS OCRs were more frequently distributed in introns and intergenic regions, except for the BM ILC2 TS OCRs, a large proportion of which was also found at promoters (Supplementary Fig. S6b). We found that ILC2 TS OCR-correlated genes, rather than commonly reduced peaks-related genes, overlapped with ILC2 TS genes including *Lztfl1*, *Klrk1*, *Smad7*, *Alox5*, and *Gzma* as representatives (Fig. 5d and Supplementary Fig. S6c).

Next, we performed integrated analysis on ILC2 TS OCRs with gene mRNA expression of tissue ILC2s (Supplementary Fig. S6d and Supplementary Data 2). We found that the change of more than 50% differentially expressed genes was in line with genome accessibility at all analyzed regions of the gene locus, indicated by upregulated genes having increased genome accessibility compared to other tissue ILC2s (Supplementary Fig. S6d, upper right quadrant of coordinate axis), whereas decreased genes showing reduced genome accessibility (Supplementary Fig. S6d, lower left quadrant of coordinate axis). Importantly, genome accessibility at the intragenic and intergenic regions had better correlation with gene mRNA expression compared with accessibility of promoter regions, as was indicated by the correlation coefficient (Supplementary Fig. S6d). Together, the data suggest that environmental factors could imprint ILC2 tissue signatures by promoting chromatin accessibility especially at non-promoter regions.

Next, motif enrichment analysis was performed on ILC2 TS OCRs (Fig. 5e and Supplementary Data 3). GATA motifs were found to be shared among all tissue ILC2 TS OCRs, whereas KLF motifs, ROR motifs, SMAD motif, and IRF motifs were enriched in BM, LI, lung, and SI ILC2 OCRs, respectively (Fig. 5e). Among representative motif-related transcription factors, we found that *Rora* and *Smad4* mRNA were expressed at a significantly higher level by the tissue ILC2s in which their motifs were enriched in TS OCRs (Fig. 5f). The mRNA of *Irf1*, *Irf2*, *Irf3* in SI ILC2s and *Smad3* in lung ILC2s showed increased expression on average but failed to reach a significant difference (Fig. 5f). The data imply that the increased chromatin accessibility may be partly but not completely caused by enhanced expression of the transcription factors.

### Ahr sustains Aiolos to prevent exhaustion of ILC2 during in vitro expansion

**Fig. 6:**
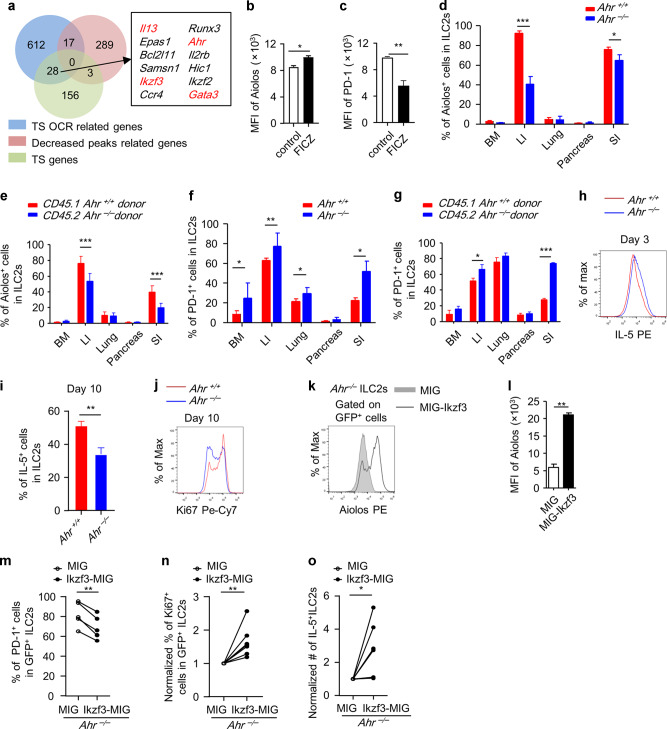
Ahr promotes Aiolos expression in ILC2s cell intrinsically. **a** LI and SI ILC2 commonly specific OCRs and differentially decreased peaks correlated genes were overlapped with LI and SI ILC2 common signature genes. **b**, **c** Purified LI ILC2s were treated with DMSO or FICZ (1 nM) for 24 h and analyzed with flow cytometry. Mean fluorescence intensity (MFI) of Aiolos (**b**) and PD-1 (**c**) gated on live cells were shown. **d**, **f** Expression of Aiolos and PD-1 in tissue ILC2s (Lin^–^GATA3^+^) from indicated organs of littermate wild-type or *Ahr*^*–/–*^ mice was analyzed by flow cytometry. **e**, **g** Mixed bone marrow cells at 1:1 ratio from CD45.2-Ahr-deficient and CD45.1-wild-type mice were transferred to half-lethally irradiated *Rag2*^*–/–*^*Il2rg*^*–/–*^ mice. Six weeks later, expression of Aiolos (**e**) and PD-1 (**g**) in ILC2s from indicated tissues was analyzed by flow cytometry. Percentages of Aiolos^+^ cells or PD-1^+^ cells gated on ILC2s from indicated origins (CD45.1^+^Lin^–^GATA3^+^ or CD45.2^+^Lin^–^GATA3^+^) are shown. Error bars are mean + SEM. Data were pooled from three to five mice of two independent experiments. **h**–**j** ILC2s were purified from the large intestine of wild-type (WT, dashed line) or *Ahr*^*–/–*^ mouse and cultured in vitro in the presence of IL-7 and IL-33. Expression of IL-5 was analyzed by flow cytometry on day 3 (**h**) or day 10 (**i**) with brefeldin A added in the last 2 h. **j** Expression of Ki67 was analyzed by flow cytometry on day 10 after culture. **k**–**o** ILC2s were expanded for 5 days and infected with retrovirus expressing MIG or Ikzf3-MIG twice in the next 2 days. GFP^+^ virus-infected cells were purified and re-plated starting with equal cell numbers for another 48 h before cells were analyzed. **k**–**n** Expression of Aiolos (**k**), PD-1 (**m**), and Ki67 (**n**) gated on GFP^+^ cells was analyzed by flow cytometry. **l** MFI of Aiolos. **o** Absolute numbers of IL-5^+^ILC2s were total numbers of ILC2 time the percentages of IL-5^+^ cells. **m**–**o** Connected lines indicate cells from same mouse and same batch of experiment. **b**–**o** Data are from two to four independent experiments. **d**–**g**, **l** Error bars are mean + SEM. LI large intestine, SI small intestine.

Genes related to LI and SI ILC2 common TS OCRs had overlap with 28 of LI and SI ILC2 common signature genes, including *Il13*, *Ikzf3*, *Gata3*, and *Ahr* (Fig. 6a). Majority of intestinal ILC2-specific OCRs were distributed at non-promoter regions (Supplementary Fig. S7a). The gene expression in intestinal ILC2s had stronger positive correlation with accessibility of intragenic and intergenic regions of the genome compared to the promoter regions, as was indicated by the correlation coefficient (Supplementary Fig. S7b). Interestingly, an Arnt:Ahr-binding motif was significantly over-presented in intestine-specific OCRs (Supplementary Data 3). Ahr has been shown to possess self-regulatory property by association with and increasing the accessibility of the *Ahr* locus.^47^ Combining the previously published ATAC-seq data using Ahr-deficient ILC2s with our analysis, we found that one of the reported Ahr-dependent cis-regulatory elements (*Ahr*-+14 kb) at the *Ahr* locus was an intestine-specific OCRs (Supplementary Fig. S7c).^37,47^

Aiolos expression in Th17 cells has been shown to be regulated by Ahr.^48^ We then tested if Ahr also regulated Aiolos expression in ILC2s. Interestingly, activation of Ahr using 6-Formylindolo[3,2-b]carbazole (FICZ), a tryptophan-derived ligand of Ahr, led to increased Aiolos expression accompanied with reduced PD-1 expression in ST2^+^ILC2s in vitro (Fig. 6b, c). In vivo, intestinal ILC2s but not ILC2s from other organs of *Ahr*^*–/–*^ mice had significantly reduced Aiolos expression compared with littermate controls (Fig. 6d). Using a BM chimeric mouse generated with mixed BM cells from Ahr-deficient or sufficient donors, we consistently found that Aiolos expression in intestinal ILC2s was significantly lower in the absence of Ahr (Fig. 6e). And PD-1 expression was increased in intestinal ILC2s from *Ahr*^*–/–*^ mice compared to controls (Fig. 6f), and in intestinal ILC2s derived from *Ahr*^*–/–*^ donors compared with wild-type donors in BM chimeric recipients (Fig. 6g). Together, the data suggest that Ahr supports Aiolos and represses PD-1 expression in intestinal ILC2s through a cell-intrinsic manner.

Ahr has been shown to suppress the maintenance of ILC2s possibly through a cell-extrinsic mechanism and to inhibit production of type 2 cytokines by cell-intrinsically inhibiting ST2 expression.^47^ Indeed, *Ahr*^*–/–*^ ILC2s expressed higher IL-5 during short-term culture (3 days) in vitro (Fig. 6h). However, *Ahr*^*–/–*^ ILC2s showed an accelerated “exhausted-like” phenotype when cultured for a longer time (10 days), manifested by reduced IL-5 expression (Fig. 6i) and decreased cell proliferation (Fig. 6j), compared with wild-type ILC2s. Importantly, overexpression of Aiolos in Ahr-deficient ILC2s suppressed PD-1 expression and reversed their “exhausted-like” phenotype by restoring cell proliferation and absolute numbers of IL-5^+^ILC2s in *Ahr*^*–/–*^ ILC2s (Fig. 6k–o). Together, the above data suggest that Ahr is critical for the long-term maintenance and IL-5 production of ST2^+^ILC2s in vitro through sustaining Aiolos, which suppresses PD-1.

### Ahr promotes *Ikzf3* transcription in ILC2s at the epigenetic level

**Fig. 7:**
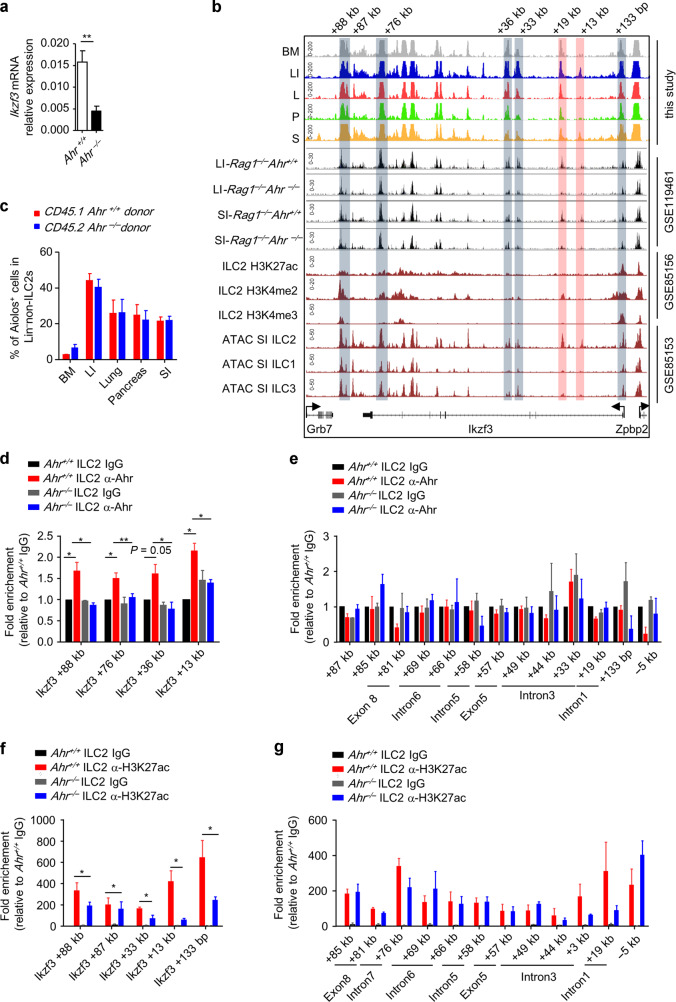
Ahr promotes the transcription of *Ikzf3* epigenetically. **a** mRNA expression of *Ikzf3* in IL-33-treated ILC2s for 7 days was analyzed by real-time RT-PCR. **b** Genome browser tracks of tissue ILC2 ATAC-seq peaks with published ATAC-seq peaks and ChIP-seq peaks at the *Ikzf3* locus. From top to bottom: ATAC-seq data on tissue ILC2s; published ATAC-seq data performed using Ahr-deficient and control ILC2s of *Rag1*^*–/–*^ mouse; published ChIP-seq data on small intestinal ILC2s; published ATAC-seq data on small intestinal ILC2s, ILC1s and ILC3s. Boxes highlight OCRs. Red boxes highlight Ahr-dependent OCRs. **c** Mixed bone marrow cells at 1:1 ratio from CD45.2-Ahr-deficient and CD45.1-wild-type mice were transferred to half-lethally irradiated *Rag2*^*–/–*^*Il2rg*^*–/–*^ mice. Six weeks later, expression of Aiolos was analyzed by flow cytometry. Percentages of Aiolos^+^ cells gated on Lin^–^non-ILC2s (CD45^+^Lin^–^GATA3^low and negative^) ILC2s from indicated origins are shown. **d**–**g** ChIP Q-PCR was performed on Ahr-deficient or wild-type (WT) ILC2s using IgG control, or α-Ahr, or α-H3K27ac antibodies. Relative enrichment of Ahr (**d** and **e**) or H3K27ac (**f** and **g**) at indicated locus marked in (**b**) normalized to IgG control group of WT-ILC2s is shown.

*Ikzf3* mRNA expression was significantly reduced in Ahr-deficient ILC2s, suggesting that Ahr may regulate *Ikzf3* at the transcription level (Fig. 7a). One intestinal ILC2-specific OCR was identified at the intron of the *Ikzf3* locus (*Ikzf3*-+13 kb) (Fig. 7b). Through analysis on the published data, we found that the accessibility of *Ikzf3*-+13 kb, together with an adjacent OCR (*Ikzf3*-+19 kb), was significantly reduced in Ahr-deficient intestinal ILC2s (Fig. 7b).^47^ This suggests that the accessibility of *Ikzf3*-+13 kb and *Ikzf3*-+19 kb is Ahr-dependent. Integrated analysis with previous data indicates that *Ikzf3*-+19 kb is a potential cis-regulatory element with H3K4me2 modification (Fig. 7b).^37^ Interestingly, both *Ikzf3*-+13 kb and +19 kb appeared to be ILC2-specific peaks compared with ILC1s and ILC3s (Fig. 7b).^37^ Consistently, decreased Aiolos expression was not observed in non-ILC2-ILCs in *Ahr*^*–/–*^ donors compared with wild-type donors in BM chimeric recipients, indicating that Ahr regulates *Ikzf3* expression cell-type specifically (Fig. 7c). A further screening on association with OCRs at the *Ikzf3* locus using ChIP Q-PCR identified that Ahr bound to +88 kb, +76 kb, +36 kb, and +13 kb loci of *Ikzf3* gene (Fig. 7d) but no other observed OCRs (Fig. 7e). Strikingly, level of H3K27ac marking active transcription was significantly decreased at +88 kb, +87 kb, +33 kb, +13 kb, and +133 bp of the *Ikzf3* locus in the absence of Ahr (Fig. 7f), consisting the *Ikzf3* promoter (+133 bp) and an Ahr-binding site (+13 kb), but no other positions (Fig. 7g). Together, the data suggest that Ahr associates with the *Ikzf3* locus and promotes the transcription of *Ikzf3* by enhancing the genome accessibility and favoring H3K27ac modification. As summarized in the working model, our findings elaborate the molecular mechanisms involved in shaping ILC2 characters by tissue microenvironment and highlight Aiolos as a potential target for treating type 2 inflammatory diseases (Supplementary Fig. S8).

## Discussion

In this study, we have interrogated the heterogeneity of tissue ILC2s shaped by tissue microenvironment at the transcriptome and epigenome level. We identified Aiolos as an intestinal ILC2-specific feature and Gal-1 as a lung/pancreas ILC2-specific feature under the steady state. Cross-tissue adoptive transfer demonstrated that the tissue signature of ILC2s was strongly shaped by microenvironment. Importantly, Aiolos could also be triggered by pro-inflammatory cytokines including IL-33 in non-intestinal ILC2s, reflecting a plasticity of ILC2 tissue-specific feature in pathologic conditions. Our study highlights the importance for verification of tissue-specific features both under the steady state and during diseases for targeting ILC2s in site-specific and disease-specific manner.

Although ILC2 tissue features may be driven by one or two dominant factors, such as IL-33, IL-1β, TL1A, and TGF-β identified in our research, a collection of both supportive and suppressive signals may cooperatively regulate ILC2 tissue signatures. Whole genome-wide screening on tissue transcriptional profiles, facilitated by ligand and receptor database in addition to antibody blockade and mouse genetic tools, will be helpful for an elucidation of factors endowing ILC2 tissue features.

We have identified Gal-1 as a lung and pancreas ILC2 signature. Gal-1 is an endogenous lectin that could be secreted to the extracellular matrix and bind to glycans bearing N-acetyllactosamine, which can be found in a variety of cell surface receptors including CD45, CD43, and CD7.^50^ Gal-1 plays complex roles and acts as a double edge sword in inflammatory responses. On the one hand, Gal-1 suppresses autoimmunity by expanding regulatory T cells, inducing tolerogenic DCs and increasing the apoptosis of Th1 and Th17 cells.^51–53^ On the other hand, a recent finding has shown that Gal-1 acts as a damage-associated molecular pattern and increases inflammation and lethality of sepsis.^54^ Except for functioning as a secretory protein, Gal-1 located intracellularly has been associated a transformed phenotype of malignant breast cells and anti-tumor capacity of T cells, suggesting a cell-intrinsic regulation by Gal-1.^55,56^ Therefore, secretory Gal-1 by ILC2 may bridge the crosstalk of ILC2 and other immune or non-immune cells in tissues. And intracellular Gal-1 may regulate maintenance and function of ILC2s through cell-intrinsic mechanisms. Considering that pancreatic ILC2s could promote anti-tumor response of CD8 T cells upon α-PD-1 treatment, it will be interesting to investigate the role of Gal-1 on pancreatic ILC2s during pancreatic cancer.^13^

We demonstrated that *Hspa1a* was negatively correlated with *Ikzf3* expression and knocking down *Ikzf3* caused upregulation of *Hspa1a*, encoding the heat shock protein Hsp70. Hsp70 exhibits essential roles in protein homeostasis by prevention of protein misfolding, facilitating stress granule disassembling and restoring protein translation, and mediating protein degradation after ubiquitination.^57–59^ Typically, Hsp70 is triggered by elevated temperature, oxidative stress, or protein aggregation.^59^ It remains to be examined whether *Ikzf3* knockdown-induced *Hspa1a* expression is due to a stressed state of the cells. Moreover, function of extracellular/secretory Hsp70 has been shown to affect CD8 cytotoxic response and tissue remodeling.^60,61^ Whether ablating *Ikzf3* could enhance extracellular Hsp70 derived from ILC2s and then affects other types of cells in tissue environments remain to be further explored.

We have shown that majority of ILC2 TS OCRs are distributed at non-promoter areas. This suggests that potential cis-regulatory elements at non-promoter regions play important roles in regulating the expression of ILC2 tissue-specific genes. In addition, our finding that ILC2 tissue features are strongly shaped by environmental cues indicates a plasticity of ILC2 functions. However, the discovery of bona fide ILC2 tissue feature, which could hardly be driven by tissue environment, remains to be explored. Integrated multi-omics analyses involving genome-wide DNA methylation sequencing may facilitate the discovery of fate-stable ILC2 tissue signatures.

As a ligand-dependent transcription factor broadly expressed by the immune and non-immune cells, Ahr has been shown to play a critical role in intestinal immunity.^62–64^ Ahr has been found to suppress the accumulation (possibly through cell-extrinsic mechanism) and function of ILC2s previously.^47^ In consistency with the conclusion of the reported research, *Ahr*^*–/–*^ ILC2s initially expressed higher IL-5 when cultured for a short time (3 days) in vitro. However, we observed that *Ahr*^*–/–*^ ILC2s had significantly defective expansion and reduced IL-5 production when cultured for a longer time (10 days). The condition of those cells was described as “exhausted-like” phenotype since it manifested hypo-function and contraction after continuous activation, which was in contrast to their homeostatic status under the steady state. Interestingly, similar phenomenon has been reported in *Cbfβ*^*–/–*^ mice, in which *Cbfβ*^*–/–*^ILC2s produce higher IL-5 under steady state whereas show “exhausted hyporesponsiveness” with decreased secretion of type 2 cytokines when cultured in vitro or during chronic inflammation in vivo.^65^ Consistent with the role of Aiolos in suppressing PD-1, “exhausted-like” *Ahr*^*–/–*^ ILC2s have reduced Aiolos expression accompanied by increased PD-1 expression. And we further showed that the exhaustion of Ahr-deficient ILC2s could be rescued by overexpression of Aiolos. Therefore, Ahr prevents ILC2s entering an “exhausted-like” state by supporting Aiolos expression. Our work provides a mechanism for regulating ILC2 exhaustion and could be enlightening for optimizing ILC2 in vitro expansion used for ILC2-based cell therapies.

From published data, we have identified two OCRs (*Ikzf3*-+13 kb and *Ikzf3*-+19 kb) at intron 1 of the *Ikzf3* locus, the accessibility of which is dependent on Ahr in ILC2s.^47^ These two sites could possibly act as Ahr-dependent enhancers driving *Ikzf3* expression in ILC2s. Moreover, *Ikzf3*-+13 kb and *Ikzf3*-+19 kb are specifically accessible in ILC2s but not in ILC1s or ILC3s,^37^ in align with the observation that Aiolos was not decreased in non-ILC2-ILCs of Ahr-deficient mice, suggesting that epigenetic regulation of *Ikzf3* by Ahr is cell-type specific. In summary, our findings suggest that integrated analysis of tissue-resident cells at transcriptome and epigenome level reveals distinct regulatory manners involving specific sets of machineries imprinted by tissue microenvironment. Interrogation of molecular features of multi-tissue ILC2s will provide tissue-specific targets for treating type 2 inflammatory diseases occurring in different organs.

## Methods

### Mice

Cas9-transgenic mice were generated through oocyte injection of genetically modified androgenetic haploid embryonic stem cells (AG-haESCs) according to the previous publications. To establish the AG-haESC line carrying constitutively expressed Cas9, first we constructed a transposon mediated Cas9 transgene vector PB-Cas9-mRFP via subcloning Cas9 cassette into a PB-mRFP backbone. Then, PB-Cas9-mRFP supplemented with PBase were transfected into AG-haESCs using Lipofectamine 3000 (Life technology) according to the manufacturer’s instruction manual. After cell culture for 1 week, mRFP positive haploid cells were enriched with flow cytometry (FACS AriaII, BD Biosciences), following with single-cell expansion. The established cell lines were selected based on their mutation efficiency and then prepared for mouse construction.

Wild-type mice were purchased from Shanghai SLAC Laboratory Animal (Shanghai, China). CD45.1 mice were purchased from the Jackson laboratory (Bar Harbor, ME). *Rag2*^*–/–*^*Il2rg*^*–/–*^ mice were purchased from Taconics Biosciences (Rensselaer, NY). *Il5*^*tm1.1(icre)Lky/J*^ (Red5) mice were purchased from the Jackson laboratory, and *Ahr*^*–/–*^ mice were generated previously.^45,66^ *Pdcd1*^*–/–*^ mice were ordered from Shanghai Model Organisms Center, Inc. The mice used in this study were C57BL/6 background and maintained in specific pathogen-free conditions. For single-cell RNA sequencing and ATAC-sequencing, 6-week-old male mice were used. In other experiments, both male and female 6–10-week-old mice were used. All animal experiments were performed in compliance with the “Guide for the Care and Use of Laboratory Animals” and approved by the institutional biomedical research ethics committee of the Shanghai Institutes for Nutrition and Health, Chinese Academy of Sciences.

### Isolation of mononuclear cells from tissues

#### Bone marrow cells were processed by flushing femur and tibia using a syringe

For isolation of mononuclear cells from lung or pancreas, tissues were dissected and blood clots, fat tissues, and bronchus were discarded. Lung or pancreas tissues were cut into pieces and digested with 5 ml RPMI 1640 medium (Thermo Fisher Scientific) containing DNase I (75 ug/ml for digestion of lung, pancreas was digested without DNase I, Sigma-Aldrich) and collagenase VIII (200 U/ml, Sigma-Aldrich) at 37 °C for 1 h (for digestion of lung) or 30 min (for digestion of pancreas). The digested tissues were homogenized by vigorous shaking and passed through a 70 μm cell strainer. Mononuclear cells were then harvested from the interphase of a 40 and 80% Percoll (GE Healthcare) gradient after a spin at 2500 rpm for 20 min at room temperature.

For isolation of large and SI lamina propria lymphocytes, tissues were dissected. Fat tissues and peyer’s patches in the SI were removed. The intestines were then cut into pieces, washed, and shaken in PBS containing 1 mM dithiothreitol for 10 min at room temperature. Intestines were incubated with shaking at 220 rpm in PBS containing 30 mM ethylenediaminetetraacetic acid (EDTA) at 37 °C for 10 min for two cycles. The tissues were then digested in 5 ml RPMI 1640 medium containing DNase I (150 ug/ml) and collagenase VIII (150 U/ml) at 37 °C in an incubator with 5% CO_2_ for 1.5 h. Mononuclear cells were then harvested from the interphase of a 40 and 80% Percoll gradient after a spin at 2500 rpm for 20 min at room temperature.

### Isolation of mononuclear cells from human samples

The study was approved by the Independent Ethics Committee of Shanghai Tongren Hospital (approval number 2019-052-01 and 2020-043-01) for obtaining human BM cells and human colon tissues. Human BM cells were obtained from the remaining materials of healthy donors subjected to medical tests aged from 30 to 65 years old. Human peripheral blood was obtained from the remaining materials of healthy donors subjected to medical tests aged from 20 to 30 years old. BM cells or peripheral blood was mixed with an equal volume of PBS and then mononuclear cells were then harvested from the interphase of Ficoll (GE Healthcare) gradient for BM cells and or the interphase of Lymphoprep™(Axis-Shield) gradient for peripheral blood after a spin at 2000 rpm for 20 min at room temperature.

Human colon samples were obtained from the intraoperative normal tissues (≥10 cm away from the tumor) collected from colorectal cancer patients with informed consent at the Department of General Surgery of Tongren Hospital. Fresh tissues were washed with pre-cold medium and the fat tissues and muscle were removed. The intestinal mucosa about 2 cm × 2 cm was cut into pieces and shaken in PBS containing 1 mM dithiothreitol for 10 min at room temperature. Then, tissue pieces were further incubated in a shaker at 220 rpm in PBS containing 30 mM EDTA at 37 °C for 10 min for two times. The tissues were then digested in 10 ml RPMI 1640 medium containing DNase I (Sigma-Aldrich, 300 ug/ml) and collagenase VIII (Sigma-Aldrich, 400 U/ml) at 37 °C in the cell culture incubator with 5% CO_2_ for 6 h. Mononuclear cells were then harvested from the interphase of a 40 and 80% Percoll gradient after a spin at 2500 rpm for 20 min at room temperature.

Human lung samples were obtained from the surgery collected from patients with lung cancer from Shanghai Chest Hospital with informed consent. Fresh lung tissues from distal regions of the tumor (>5 cm) were cut into pieces and digested with DNase I (Sigma-Aldrich, 350 ug/ml) and collagenase VIII (Sigma-Aldrich, 112.5 U/ml) at 37 °C for 45 min. Cells harvested from the digestion were directly used for analysis.

### Flow cytometry and cell sorting

Dead cells were stained with live and dead violet viability kit (Invitrogen) and were gated out in cell sorting and cell analysis. Anti-mouse CD16/32 antibody was used to block the non-specific binding to Fc receptors before all surface stainings. Antibodies used for regular flow cytometry are listed in Supplementary Data 4. For detection of nuclear factor, cells were fixed and permeabilized using a Mouse Regulatory T Cell Staining Kit (Thermo Fisher Scientific). For detection of protein expression in cytoplasm, BD Cytofix/Cytoperm™ kit was used. For detection of 4-1BB, AREG, GM-CSF, and IL-5, cells were stimulated by PMA (50 ng/ml, Sigma-Aldrich) and ionomycin (500 ng/ml, Sigma-Aldrich) for 4 h, and brefeldin A (2 ug/ml, Sigma-Aldrich) was added for the last 2 h before cells were harvested for analysis. Unless used for sorting, cells were collected by using Gallios flow cytometer (Beckman Coulter) and analyzed with FlowJo software (TreeStar). For cells sorted for scRNA-seq analysis, ILC2s from BM, LI, lung, and pancreas were sorted as Lin^–^ST2^+^CD25^+^ cells. And ILC2s from SI were sorted as Lin^–^Thy1^low^CD45^+^KLRG1^+^ cells. For cells sorted for ATAC-seq analysis, ILC2s from BM were sorted as Lin^–^ST2^+^CD25^+^ cells. ILC2s for LI, lung, and pancreas were sorted as Lin^–^CD45^+^CD127^+^ST2^+^CD25^+^. And ILC2s from SI were sorted as Lin^–^CD127^+^Thy1^low^CD45^+^KLRG1^+^ cells. BM ILC2s were sorted using BD FACSARIA III (BD biosciences). And ILC2s of other tissues were sorted using Moflo Astrios (Beckman). For mouse cell sorting used for sequencing, lineage markers were CD3, B220, CD11b, FcεRI, and CD11c. In all other mouse experiments, lineage markers were CD3, B220, CD11b, and CD11c. In all human experiments, lineage markers were CD3, CD5, CD19, CD11b, CD11c, CD14, and FcεRI.

### scRNA-seq analysis

ILC2s sorted from five tissues of wild-type male mice were used for scRNA-seq. Viability of cells was more than 95% before cells were loaded on the Chromium Single Cell Controller (10X Genomics). Libraries were generated using the Chromium Single Cell 3′ Reagents Kits (v2 chemistry; 10X Genomics) following manufacturer’s protocol.^67^ The obtained libraries were sequenced on Illumina Hiseq X Ten system. Libraries of ILC2s from lung, LI, and SI were generated together in one batch of experiment from ten mice pooled together, and libraries of ILC2s from BM, pancreas were generated in another batch of experiment from ten mice pooled together. Two rounds of sequencing were performed on each library of ILC2s from every tissue to obtain abundant reads and pooled for subsequent analysis. Cell Ranger software (v2.2.0, 10X Genomics) was used to perform demultiplexing, alignment and counting. The reads were aligned to the mm10 genome. Further quality control was performed using Scater package:^68^ (1) cells with fewer than 10^2.5^ UMIs, or with over 10% UMIs derived from mitochondrial genome (ENSMUSG00000064357,ENSMUSG00000064370,ENSMUSG00000064341,ENSMUSG00000064363,ENSMUSG00000065947,ENSMUSG00000064367,ENSMUSG00000064368,ENSMUSG00000064354,ENSMUSG00000064351,ENSMUSG00000064345,ENSMUSG00000064356,ENSMUSG00000064358,ENSMUSG00000064360), were removed. (2) Genes failed to be detected with a minimum total read counts of five in at least two cells were filtered. ScRNA-seq analysis was performed using Seurat package (version 2 and 3) in R (version 3.6.1).^69,70^ Briefly, counts were normalized using LogNormalize function. Then, FindViariableGenes, RunPCA, and JackStraw functions were used to perform linear dimensional reduction based on statistically significant principal components. FindClusters, RunTSNE, and FindAllMarkers were used to identify the cell clusters, visualize cluster, and look for differentially expressed genes. Contaminated clusters with representative signature genes for ILC3s (*Il22, Rorc, Ccr6, Klrb1c*), B cells (*Jchain, Mzb1, Ebf1, Cd79a*), T cells (*Cd28, Cd3e, Cd8a*), endothelial cells (*Thbd, Cd93*), NK cells (*Klrb1c*), myeloid cells (*Lyz2, Itgam, Itgax*), and Group 1 ILCs (*Tbx21, Ifng*) were filtered from analysis. Two rounds of filters were performed for LI and pancreas ILC2s. MergeSeurat was used to aggregate quality-filtered ILC2 single-cell data from all tissues. Cell counts of tissue ILC2s were downsampled to 2734 cells according to sample with fewest cell counts (Lung ILC2s). Hierarchically, proximity analysis was performed using PlotClusterTree function. Differentially expressed genes in clusters were identified using “1 versus 1” comparison between all clusters as one strategy and “1 versus all (other clusters as a bulk population)” as another strategy. Large and small intestinal ILC2 common signatures were obtained by overlapping large and small intestinal ILC2 differentially expressed genes compared to each of the other three tissue ILC2s (BM, lung, and pancreas ILC2s) through a “1 versus 1” method separately. Gene ontology analysis was performed using the “Gene Ontology Resource” (http://geneontology.org).

### Analysis of mRNA expression by real-time RT-PCR

RNA was isolated with Trizol reagent (Invitrogen). cDNA was synthesized using GoScript™ Reverse Transcription kit (Promega). Real-time PCR was performed using FastStart Universal SYBR Green Master (Roche Mannheim) and reactions were run with QuantStudio 7 software (Thermo Fisher Scientific). The results were displayed as relative expression values normalized to *Actb* (for mouse) or GAPDH (for human) mRNA expression. Primers used for real-time RT-PCR are listed in Supplementary Data 5. Heatmaps based on Log_10_(fold change) of relative expression were generated using HemI 1.0.

### Gene correlation analysis at the single-cell level

Clusters suspicious to be affected by enzymatic digestions in LI ILC2s were further removed for gene correlation analysis at the single-cell level.^71,72^ “Single-cell correlation” analysis was based on normalized counts in each cell at the single-cell level.^73^ Cells with no detected *Ikzf3* expression was filtered from analysis. Significantly correlated genes in expression were identified according to *p* value of Pearson correlation analysis (*p* < 0.05).

### ATAC-seq and analysis and integrated analysis with scRNA-seq

Biological duplicates of 50,000 ILC2s from five tissues of wild-type male mice (16 mice were pooled for BM, pancreas and lung ILC2, 10 mice were pooled for SI and LI ILC2) were sorted by flow cytometry and were subjected to ATAC-seq analysis. Library preparation was performed using TruePrepTM DNA Library Prep Kit V2 for Illumina^®^ kit from Vanzyme (Nanjing, China) based on a previously published method.^74^ 1.2 × Agencourt AMPure XP beads (Beckman Coulter) were used to purify libraries for sequencing; 150 paired-end sequencing was performed with Illumina HiSeq X Ten.

ATAC-seq raw sequence reads were initially processed by FastQC (version 0.11.9) for quality control, and then adapter sequences and poor quality reads were removed. Quality-filtered reads were then mapped to mouse genome (mm10) using Bowtie2 (version 2.2.9), and only uniquely mapped reads were kept. Sam files were converted to Bam format using Samtools (version 1.8). Peak calling was done using MACS (2.1.1) with an initial threshold *q* value of 0.01 as cutoff. Only peaks repeatedly present in biological repeats were considered as “genuine peaks.” Overlapped tissue ILC2 ATAC-seq peaks were identified by first quantifying peak signal using bedtools (version 2.29.2) multicov. “MergePeaks” of HOMER (version 4.1.5) was used to define common peaks. Then, differentially expressed peaks were analyzed using DESeq2. Differentially expressed peaks significantly higher or lower (*p* < 0.05) in one tissue ILC2s compared to other tissue ILC2s through a 1 versus 1 comparison mode were overlapped. And the overlapped peaks were defined as ILC2 TS OCRs or “commonly decreased peaks.” To look for large and small intestinal ILC2 common OCRs, differentially expressed peaks were first identified in large intestinal or small intestinal ILC2s compared to each of the other three tissue ILC2s, respectively, and then overlapped peaks were intestinal ILC2-specific OCRs. Visualization of read count data was performed by converting raw bam files to bigwig files using IGV tools. Motif enrichment analysis was performed using HOMER.

Integrated analysis of ATAC-seq and scRNA-seq data was performed similar a previously published method.^75^ The correlation of mRNA expression with chromatin accessibility was performed using Log_2_(fold change) (Log_2_Fc) of mRNA expression with average Log_2_Fc of accessibility of peaks in promoter, intragenic (5’UTR + 3’UTR + exon + intron) or intergenic regions annotated by HOMER. Only genes with significantly differential mRNA expression for more than 1.2 fold in tissue ILC2s through a “1 versus all comparison” were analyzed. Average Log_2_Fc of genome accessibility was calculated by sum of Log_2_Fc in accessibility of all observed peaks divided by the number of observed peaks at promoter, intragenic, or intergenic regions of correlated genes, regardless of significance in differential expression. Log_2_Fc of gene expression was analyzed by Seurat through “1 versus all” comparison. Specifically, for LI and SI common ILC2 features, the average Log_2_Fc (mRNA expression fold and fold of genome accessibility were both processed in this way) was obtained by following calculation. First, Log_2_Fc of LI or SI ILC2 over each of the other three tissue ILC2s (BM, lung, and pancreas ILC2s) through a “1 versus 1” method was summed and divided by 3 to obtain Log_2_Fc(LI/BM–lung–pancreas) and Log_2_Fc(SI/BM–lung–pancreas). Then, the above 2 Log_2_Fc values were further added up and divided by 2. Pearson correlation analysis was performed with GraphPad Prism 5.0.

### In vitro culture of mouse ILC2s and treatment with cytokines

ILC2s (Lin^–^ST2^+^CD25^+^cells) were purified from the LI of wild-type mice, *Ahr*^*–/–*^ or *Pdcd1*^*–/–*^ mice and cultured in 96-well flat-bottom plates in RPMI 1640 complete medium containing 10% fetal bovine serum (Gibco), 2 mM glutamine (Gibco), 50 uM of β-mercaptoethanol, non-essential amino acid (Gibco), 333 U/ml of penicillin (Gibco), and 333 µg/ml of streptomycin (Gibco). For all in vitro experiments used in this study, concentration for IL-7 (Peprotech) and IL-33 (BioLegend) were 10 ng/ml.

### Sorting, culture, and siRNA treatment of human ILC2s

Human ILC2s were sorted as (Lin^–^CD45^+^CD127^+^CRTH2^+^) by flow cytometry from PBMC mixed from ten individuals as one biological sample. Cells were then cultured in 96-well flat-bottom plates with 10,000 cells per well with the same medium recipe used for culturing mouse ILC2s. Cells were treated with recombinant human (rh)IL-2(20 ng/ml, Peprotech) and rhIL-7(20 ng/ml, Peprotech) in the presence or absence of rhIL-33(50 ng/ml, Peprotech) for 5 days. For ablation of *IKZF3*, siRNA(3pmol) targeting *IKZF3* or control siRNA were transfected to purified human ILC2s cultured with IL-2, IL-7, and IL-33 for 3 days using Lipofectamine™ 3000 reagent (Thermo Fisher Scientific). mRNA and protein expression of Aiolos and IL-5 was analyzed 2 days after transfection.

### In vitro retroviral infection and treatment of mouse ILC2s with α-PD-1

MSCV-LTRmiR30-PIG (LMP) is a retroviral vector designed for the dual expression of GFP and short hairpin RNAs (shRNA) (Open Biosystems). Retro-gRNA-eGFP plasmid was a gift from Christophe Benoist & Diane Mathis (Addgene plasmid #116926; RRID:Addgene_116926).^76^ MIG plasmid was used previously.^77^ MIG-Ikzf3 was generated by cloning mouse *Ikzf3* cDNA to MIG vector. LMP-Ikzf3shRNA#1, LMP-Ikzf3shRNA#2 were generated by ligation of synthesized DNA sequences targeting *Ikzf3* (Supplementary Data 5) to the LMP vector. Ikzf3-sgRNA#1 and Ikzf3-sgRNA#2 were generated by cloning synthesized DNA sequenced targeting *Ikzf3* to the Retro-gRNA-eGFP vector (Supplementary Data 5). Phoenix cells were transfected with retroviral plasmids and the packaging plasmid 10A1 using polyethylenimine (PEI, Polysciences). Viral supernatant was collected after transfection.

For retroviral infection of ILC2s (using LMP, LMP-Ikzf3shRNA#2, MIG or MIG-Ikzf3), purified large intestinal ST2^+^ILC2s from wild-type, or *Ahr*^*–/–*^, or *Pdcd1*^*–/–*^ mice were expanded with IL-7 and IL-33 for 5 days. On day 5, spin-infection was performed using cells in 96-well flat-bottom plates in 200 ul of virus supernatant containing 8 μg/ml polybrene (Sigma-Aldrich), IL-7 (10 ng/ml), and IL-33 (10 ng/ml) at 2500 rpm for 1.5 h at 30 °C and cultured with an additional 2 h in an incubator with 5% CO_2_. Virus supernatant was then replaced with complete medium with IL-7 and IL-33. The retroviral transduction was repeated 24 h later. The infected GFP^+^ cells were sorted 24 h after the second spin-infection and re-plated with an equal number of 30,000 cells/well in each group. Cells were treated with or without α-PD-1 (J43, 10 ug/ml, Bioxcel) or hamster IgG (10 ug/ml, BioLegend) for 48 h before analysis. Brefeldin A (2 ug/ml) was added for the last 2 h of culture for detection of IL-5 by flow cytometry.

In the *Ikzf3*-knockdown experiment, mRNA expression of *Hspa1a* in purified GFP^+^ cells was analyzed 24 h after retroviral infection. For detection of *Gata3*, *Rora*, *Il5*, *Pdcd1*, and *Areg* mRNA expression, GFP^+^ cells were harvested 72 h after retroviral infection.

### Adoptive transfer of cells to *Rag2*^*–/–*^*Il2rg*^*–/–*^ mice

For repletion of ILC2s from different tissues to *Rag2*^*–/–*^*Il2rg*^*–/–*^ mice, BM ILC2s, LI ILC2s, lung ILC2s, and SI ILC2s were sorted by flow cytometry and cultured with IL-7 and IL-33 for 4 days. A total of 2 × 10^5^ cells were harvested and transferred to *Rag2*^*–/–*^*Il2rg*^*–/–*^ mice, which were immediately intraperitoneally injected with 500 ng IL-33 for 4 consecutive days. Mice were sacrificed for analysis 4 weeks after transfer.

For construction of BM chimeric mice, BM cells were harvested from age and gender-matched CD45.2-*Ahr*^*–/–*^and CD45.1-wild-type mice and mixed at a ratio of 1:1. A total of 5 × 10^6^ mixed BM cells were intravenously injected into *Rag2*^*–/–*^*Il2rg*^*–/–*^ mice irradiated with 550 rads with 4-h interval. Host mice were sacrificed for analysis 6 weeks after transfer.

For adoptive transfer of *Ikzf3*-knockdown ILC2s, 2 × 10^5^ ILC2s infected with control virus or LMP-Ikzf3shRNA#2 virus were intravenously injected into *Rag2*^*–/–*^*Il2rg*^*–/–*^ mice. For detection of IL-5, PD-1, and AREG expression in ILC2s, mice were sacrificed 4 weeks after transfer for analysis. For analysis of eosinophil infiltration, *Rag2*^*–/–*^*Il2rg*^*–/–*^ mice reconstituted with ILC2s infected with control virus or LMP-Ikzf3shRNA#2 virus were treated with 500 ng IL-33 for 4 consecutive days and sacrificed for analysis on day 5.

### Chromatin immunoprecipitation (ChIP) assay

LI ILC2s (Lin^−^ST2^+^CD25^+^ cells) were purified from littermate *Ahr*^*–/–*^ and *Ahr*^*+/+*^ mice and cultured with IL-7 and IL-33 for 7 days. For ChIP of Ahr, cells were treated with FICZ (200 nM) for 4 h before cells were harvested. Cells were cross-linked with 1% formaldehyde for 10 min and the reaction was stopped by adding glycine solution of a final concentration of 0.125 M for 10 min. Chromatin was sheared by sonication with Vibra-Cell™ VCX150 (SONICS) (20% of power, 10 s on and 15 s off for 36 cycles) to 500 bp, and immune-precipitated with anti-Ahr (Enzo Life Science), or anti-H3K27ac (Abcam), or rabbit IgG (Abcam). The target DNA was purified by Magna ChIP^TM^ Protein A Magnetic Beads (Sigma, 16-661X) and the eluted DNA was extracted and used for real-time PCR analysis. Primers used for quantitative PCR following ChIP are listed in Supplementary Data 5.

### Statistical methods and *Z*-score

The following statistical analysis methods were performed using GraphPad Prism software (version 5.0 and 8.0). Statistical analyses were performed with two-tailed paired Student’s *t*-test, except that Supplementary Fig. S2e, g was performed with two-tailed unpaired Student’s *t*-test and Fig. 7d–g was performed with one-tailed paired Student’s *t*-test. Paired data were cells from one mouse from the same batch of experiment, or littermate pairs of mice. Data from such experiments are presented as means + SEM; *p < 0.05 was considered statistically significant; **p < 0.01; ***p < 0.001.

For where heatmaps were made with standard score (*Z*-score), the standard score of a raw score *x* is *Z* = (*x* – *μ*)/*σ*. *μ* is the mean of the expression and *σ* is the standard deviation of the expression.

## Supplementary information

Supplementary Information

Supplementary Data 1

Supplementary Data 2

Supplementary Data 3

Supplementary Data 4

## Data Availability

The data files for the tissue ILC2 scRNA-seq in this study were deposited in the National Omics Data Encyclopedia (NODE) database in experiment OEX003341 under the project OEP000918, and were also deposited in GEO: GSE153748. The accession number for ILC2 ATAC-seq data in this study is GEO: GSE150179. Tissue ILC2 sub-cluster signature genes and tissue ILC2-specific OCRs were uploaded as processed data in GEO153748 and GSE150179, respectively. ATAC-seq data and ChIP-seq data for small intestinal ILCs were published previously with accession number GSE85153 and GSE85156.^37^ ATAC-seq data on ILC2s from Ahr-deficient and control mice of the *Rag1*^*−/−*^ background were published previously with accession number GSE119461.^47^
