# Supplementary material for: Tissue signals imprint Aiolos expression in ILC2s to modulate type 2 immunity
Source: Mucosal Immunol. 2021 Aug 4;14(6):1306–22. doi: 10.1038/s41385-021-00431-5 (PMC8528704; doi:10.1038/s41385-021-00431-5)
Supplement: Supplementary file 1 — Supplementary Information [file 41385_2021_431_MOESM1_ESM.pdf]

Tissue signals imprint Aiolos expression in ILC2s to modulate type 2 immunity

Qiu et al.
Supplementary Information

- Supplementary figures and legends
- Supplementary table 1
- Supplementary Data legends

Figure S1

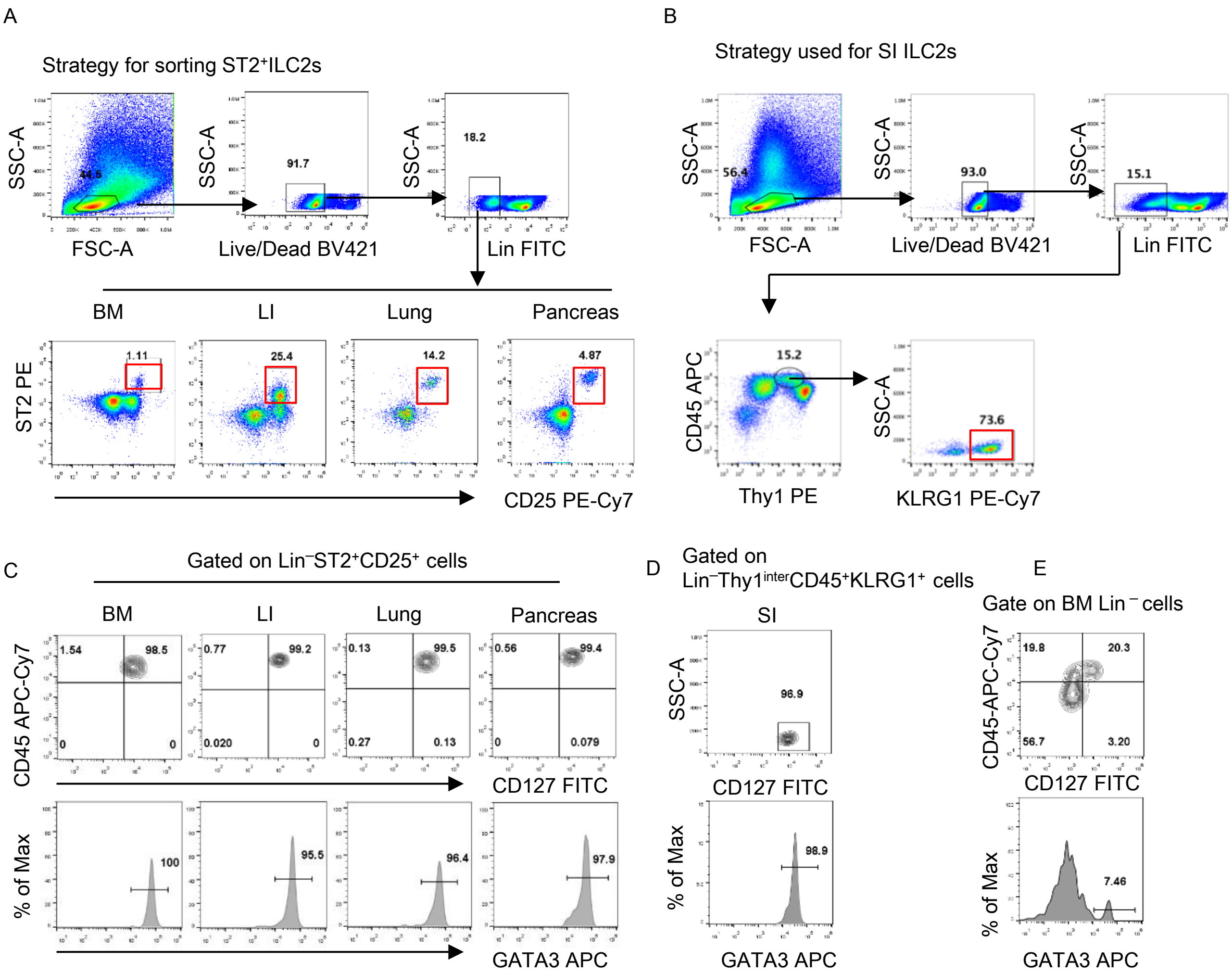

Figure S1 Sorting strategy of tissue ILC2s used for single cell sequencing

Mononuclear cells were isolated from indicated tissues. (A and B) Sequential gating strategies for sorting ILC2s from indicated tissues (highlighted in red boxes) for single cell transcriptome sequencing are shown. (C) Expression of CD45, CD127 and GATA3 gated on Lin$^-$ST2$^+$CD25$^+$ cells from indicated tissues is shown. (D) Expression of CD127 and GATA3 gated on Lin$^-$Thy1^{low}CD45$^+$KLRG1$^+$ cells from small intestine is shown. (E) Bone marrow total Lin$^-$ cells were used as control to set gates for CD127, CD45 and GATA3 in (C) and (D). (F) Cells with unmet qualities were identified by Scater and filtered from downstream analysis. Cell numbers before and after filtering are shown. BM, bone marrow; LI, large intestine; SI, small intestine.

Figure S2

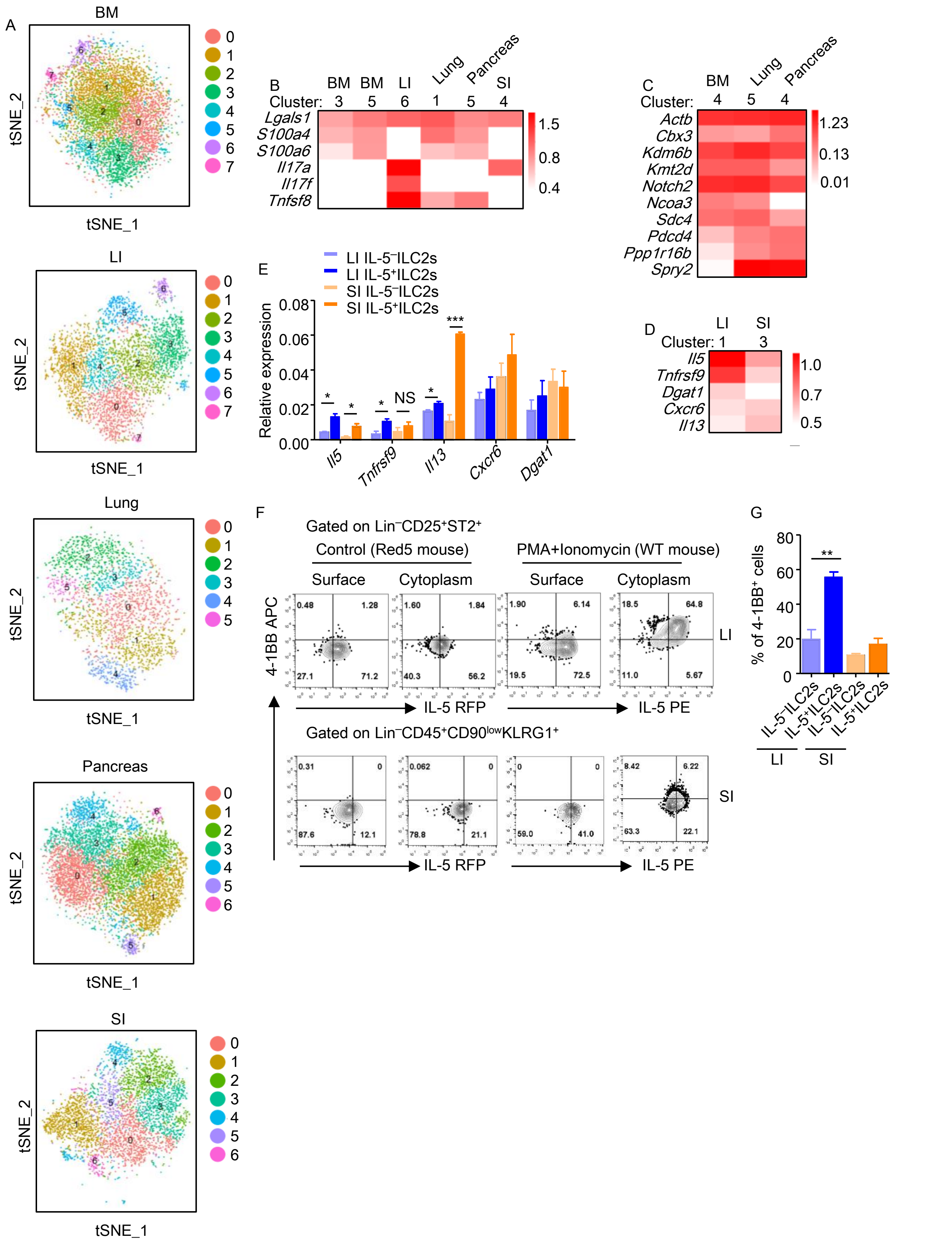

A
BM
LI
Lung
Pancreas
SI
tSNE_1
tSNE_2
0
1
2
3
4
5
6
7
B
Cluster:
BM 3
BM 5
LI 6
Lung 1
Pancreas 5
SI 4
Lgals1
S100a4
S100a6
Il17a
Il17f
Tnfsf8
1.5
0.8
0.4
C
Cluster:
BM 4
Lung 5
Pancreas 4
Actb
Cbx3
Kdm6b
Kmt2d
Notch2
Ncoa3
Sdc4
Pdcd4
Ppp1r16b
Spry2
1.23
0.13
0.01
D
Cluster:
LI 1
SI 3
Il5
Tnfrsf9
Dgat1
Cxcr6
Il13
1.0
0.7
0.5
E
LI IL-5⁻ILC2s
LI IL-5⁺ILC2s
SI IL-5⁻ILC2s
SI IL-5⁺ILC2s
Relative expression
0.08
0.06
0.04
0.02
0.00
*
NS
***
Il5
Tnfrsf9
Il13
Cxcr6
Dgat1
F
Gated on Lin⁻CD25⁺ST2⁺
Control (Red5 mouse)
PMA+Ionomycin (WT mouse)
Surface
Cytoplasm
4-1BB APC
0.48
1.28
27.1
71.2
1.60
1.84
40.3
56.2
1.90
6.14
19.5
72.5
18.5
64.8
11.0
5.67
LI
IL-5 RFP
IL-5 PE
Gated on Lin⁻CD45⁺CD90lowKLRG1⁺
0.31
0
87.6
12.1
0.062
0
78.8
21.1
0
0
59.0
41.0
8.42
6.22
63.3
22.1
SI
G
% of 4-1BB⁺ cells
80
60
40
20
0
**
IL-5⁻ILC2s
IL-5⁺ILC2s
IL-5⁻ILC2s
IL-5⁺ILC2s
LI
SI

Figure S2 Sub-clusters with similar features shared among tissue ILC2s

scRNA-seq data on tissue ILC2s were analyzed with Seurat. (A) Clustering of tissue ILC2s were analyzed with t-SNE algorithm. (B, C and D) Heatmaps show tissue sub-clusters with shared features among ILC2s from indicated tissues. White blocks indicate the gene was not detected as a cluster specific feature in indicated tissues. Scale bar indicates Log_2(fold) of gene expression in indicated cluster over other ILC2s within the same tissue. (E) Expression of indicated genes from FACS sorted IL-5-RFP$^+$ or IL-5-RFP$^-$ ILC2s (LI ILC2s were Lin$^-$ST2$^+$CD25$^+$, SI ILC2s were Lin$^-$Thy1^{low}CD45$^+$KLRG1$^+$) of Red5 mouse was analyzed by real-time RT-PCR. (F and G) SI or LI LPLs were stimulated with or without PMA and ionomycin. Surface or intracellular 4-1BB was detected by flow cytometry. IL-5 expression was indicated by IL-5-RFP (Red5 mouse was used) or intracellular staining. (G) Percentages of cytoplasmic 4-1BB expression gated on indicated cell populations from wild-type intestinal LPLs stimulated with PMA and ionomycin in (F) are shown. (E-G) Data are from four independent experiments, Error bars are mean+SEM. BM, bone marrow; LI, large intestine; SI, small intestine; LPL, lamina propria lymphocyte.

Figure S3

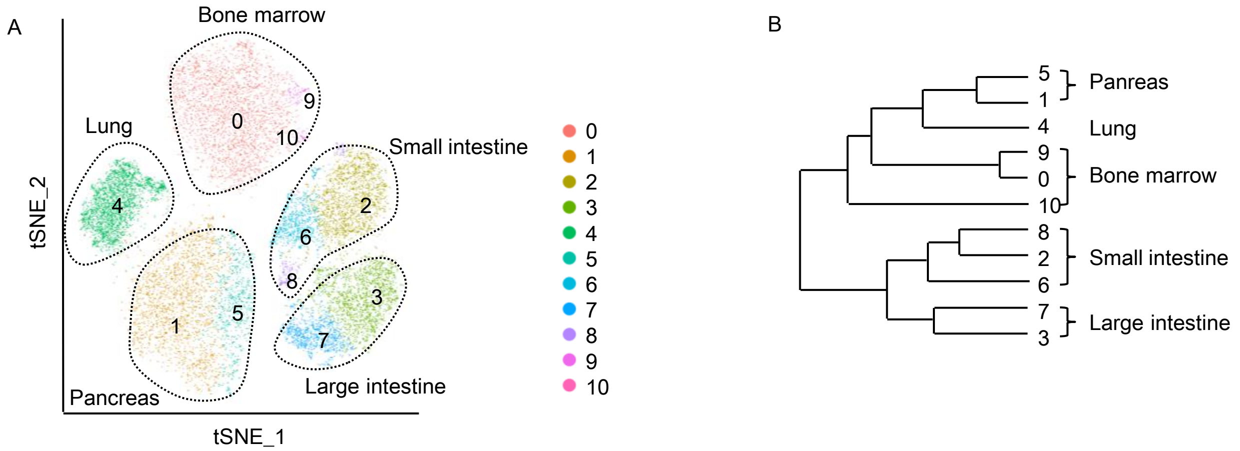

Figure S3 Comparative analysis of ILC2 scRNA-seq datasets on cluster features of tissue ILC2s

(A and B) Clustering on single cell data from tissue ILC2s (A), and hierarchically proximity analysis (B) on defined clusters from ILC2s of indicated tissues were performed using Seurat.

Figure S4

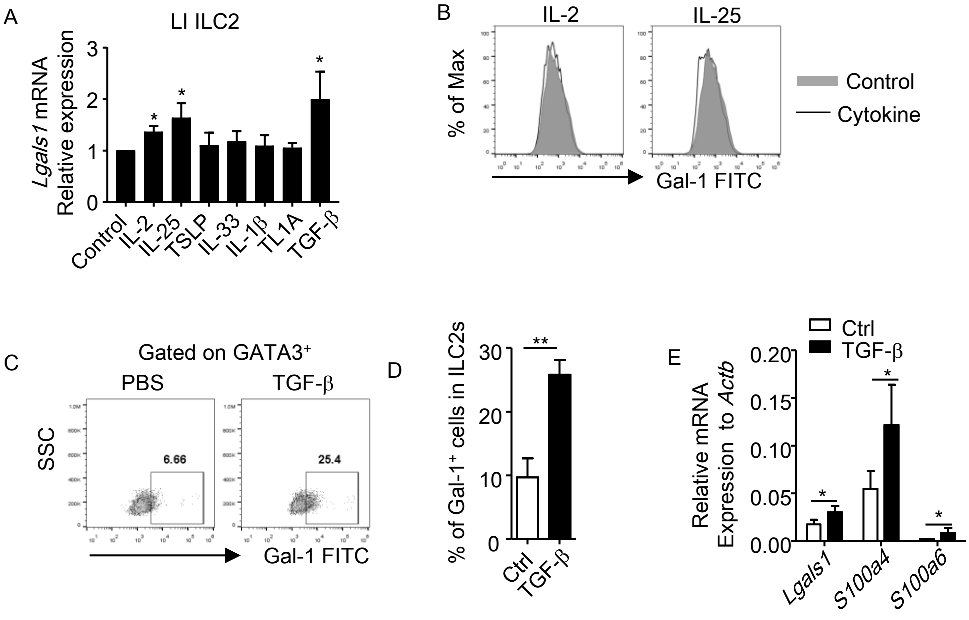

Figure S4 TGF-β induces Gal-1 expression in intestine ILC2s

Purified LI ILC2s ($Lin^{-}ST2^{+}CD25^{+}$ cells) were treated with indicated cytokines in the presence of IL-7 (10ng/ml) for 24h. Concentration for all cytokines were used at 10ng/ml except for TGF-β (1ng/ml), TL1A (100ng/ml) and TSLP (20ng/ml). (A) mRNA expression of *Lgals1* was analyzed by Q-PCR. (B and C) Expression of Gal-1 was analyzed by flow cytometry. (D) Percentages of Gal-1 expression in ILC2s are shown. (E) mRNA expression of indicated genes was analyzed by real-time PCR. (A-E) Error bars are mean ± SEM. BM, bone marrow; LI, large intestine; SI, small intestine; P, pancreas; L, lung. Data are representative of 2-4 independent experiments.

Figure S5

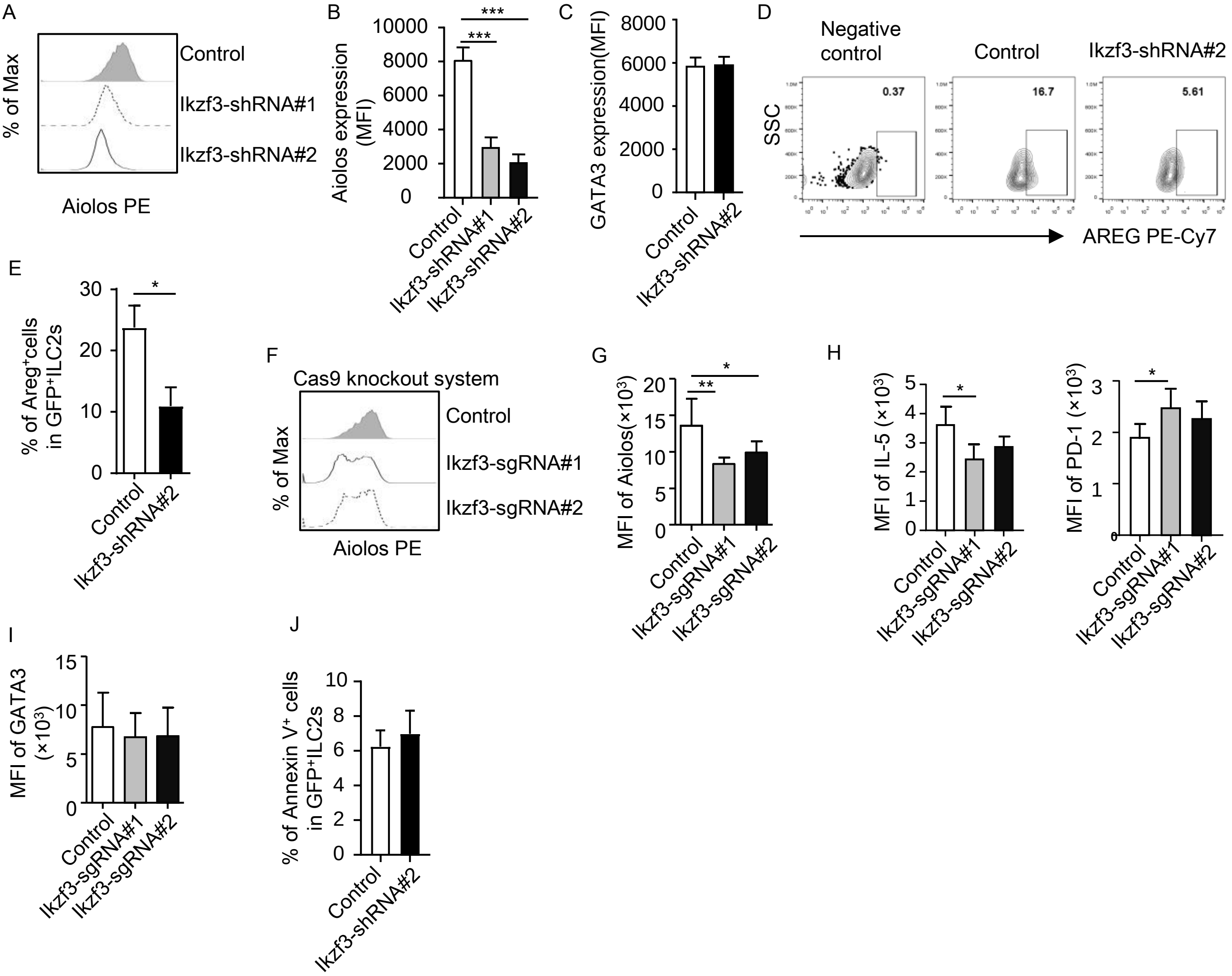

Figure S5 Aiolos sustains IL-5 expression by inhibiting PD-1 expression

(A-C, J) ILC2s purified from large intestine of wild-type mice were infected with retrovirus expressing MSCV-LTRmiR30-PIG (LMP) or *Ikzf3*-shRNA#1, or *Ikzf3*-shRNA#2. (F-I) ILC2s purified from large intestine of Cas9 transgenic mice were infected with retrovirus expressing Retro-gRNA-eGFP or *Ikzf3*-sgRNA#1 or *Ikzf3*-sgRNA#2 (all containing GFP as reporter). (A-C, F-I) Cells were analyzed 48h after the second virus infection. (J) GFP^+ILC2s were purified, re-plated with an equal cell number and analyzed 48h later. Percentages of apoptotic cells (Annexin-V^+) gated on GFP^+ cells were analyzed by FACS. (A-C, F-I) Expression of Aiolos, GATA3, IL-5 or PD-1 gated on GFP^+ ILC2s were analyzed by flow cytometry. Representative FACS plots (A and F) or statistics for mean fluorescence intensity (MFI) (B, C, G-I) of indicated protein gated on GFP^+ cells were shown. (D and E) LI ILC2s infected with control virus expressing LMP or virus expressing Ikzf3-shRNA#2 were transferred to *Rag2*$^{-/-}$*Il2rg*$^{-/-}$ *mice.* 4 weeks after transfer, LI LPLs were isolated and analyzed. Expression of AREG gated on Lin^-GFP^+ cells from host mice was analyzed by flow cytometry. Lin^+ cells from host mice were used as a negative control for gating $AREG^+$ cells. (A-I) Data are representative of 2-5 independent experiments. Error bar represents mean+SEM for bar graphs.

Figure S6

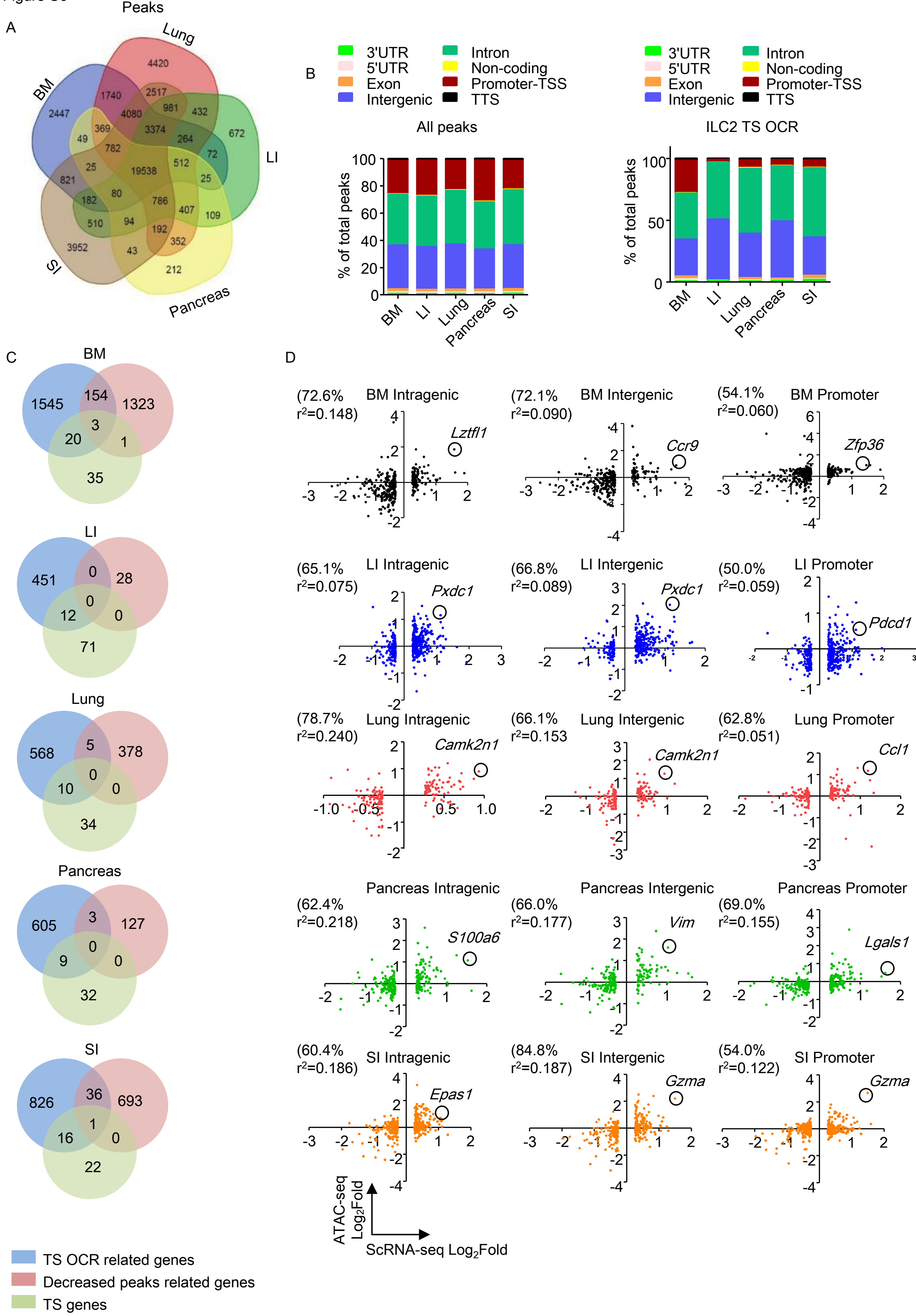

A
Peaks
Lung
BM
LI
SI
Pancreas
B
3'UTR
5'UTR
Exon
Intergenic
Intron
Non-coding
Promoter-TSS
TTS
All peaks
ILC2 TS OCR
% of total peaks
C
BM
LI
Lung
Pancreas
SI
D
BM Intragenic
BM Intergenic
BM Promoter
LI Intragenic
LI Intergenic
LI Promoter
Lung Intragenic
Lung Intergenic
Lung Promoter
Pancreas Intragenic
Pancreas Intergenic
Pancreas Promoter
SI Intragenic
SI Intergenic
SI Promoter
ATAC-seq Log2Fold
ScRNA-seq Log2Fold
TS OCR related genes
Decreased peaks related genes
TS genes

Figure S6 Accessibility of OCRs in tissue ILC2s positively correlates with gene expression
ATAC-seq data of tissue ILC2s was analyzed. (A) Venn gram shows overlapped genuine peaks identified in ILC2s from differential tissues. (B) Percentages of all identified peaks or ILC2 TS OCRs belonging to indicated peak types among total number of peaks are shown. (C) ILC2 tissue specific OCRs and commonly decreased peaks correlated genes were overlapped with ILC2 tissue signature genes. (D) Correlation analyses were performed on fold of ILC2 TS gene expression and the average fold in accessibility of OCRs distributed at promoters, intragenic regions (introns, exons, 5'UTRs and 3'UTRs) and intergenic regions. Each dot represents one gene. Representative genes were highlighted with circles. Percentages in the parenthesis indicate proportions of genes distributed in the upper right plus lower left quadrants among total analyzed genes. R^2 represents squared value of Pearson correlation coefficient. P<0.0001. BM, bone marrow; LI, large intestine; SI, small intestine; P, pancreas; L, lung.

Figure S7

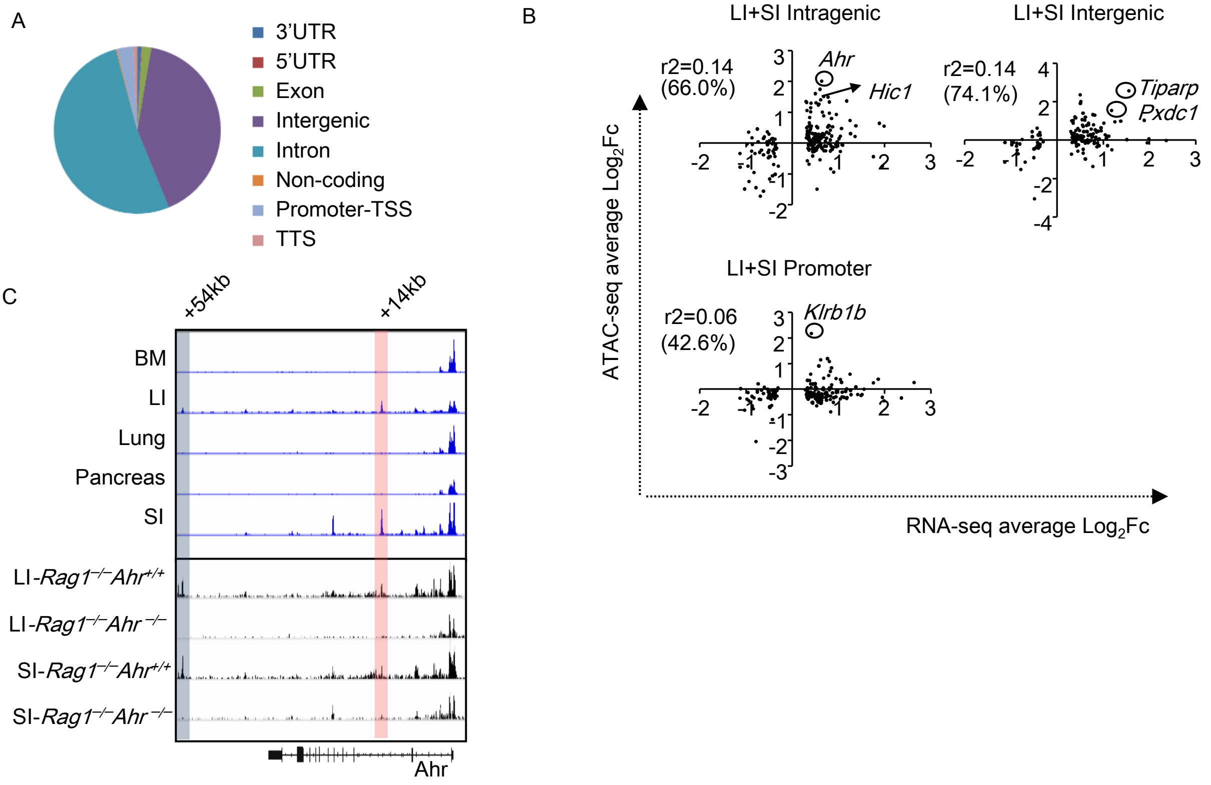

Figure S7 Ahr supports Aiolos expression in the gut with a cell-type specific manner
(A-C) ATAC-seq data of tissue ILC2s was analyzed. (A) Pie chart shows the percentages of LI and SI ILC2 commonly specific OCRs belonging to indicated peak types among total number of peaks. (B) Correlation analyses were performed on Log_2(Fold change)(Log_2Fc) of LI and SI ILC2 commonly specific gene expression and the average Log_2Fc in accessibility of OCRs distributed at promoters, intragenic regions (introns, exons, 5'UTRs and 3'UTRs) and intergenic regions. Each dot represents one gene. Percentages in the parenthesis indicate proportions of genes distributed in the upper right plus lower left quadrants among total analyzed genes. R^2 represents squared value of Pearson correlation coefficient. P<0.0001. (C) Merged visualization of tissue ILC2 ATAC-seq peaks with published ATAC-seq peaks at the Ahr locus. Top 5 rows: ATAC-seq data on tissue ILC2s; bottom 4 rows: published ATAC-seq data performed using Ahr-deficient and control ILC2s of *Rag1*$^{-/-}$ mouse.

Figure S8

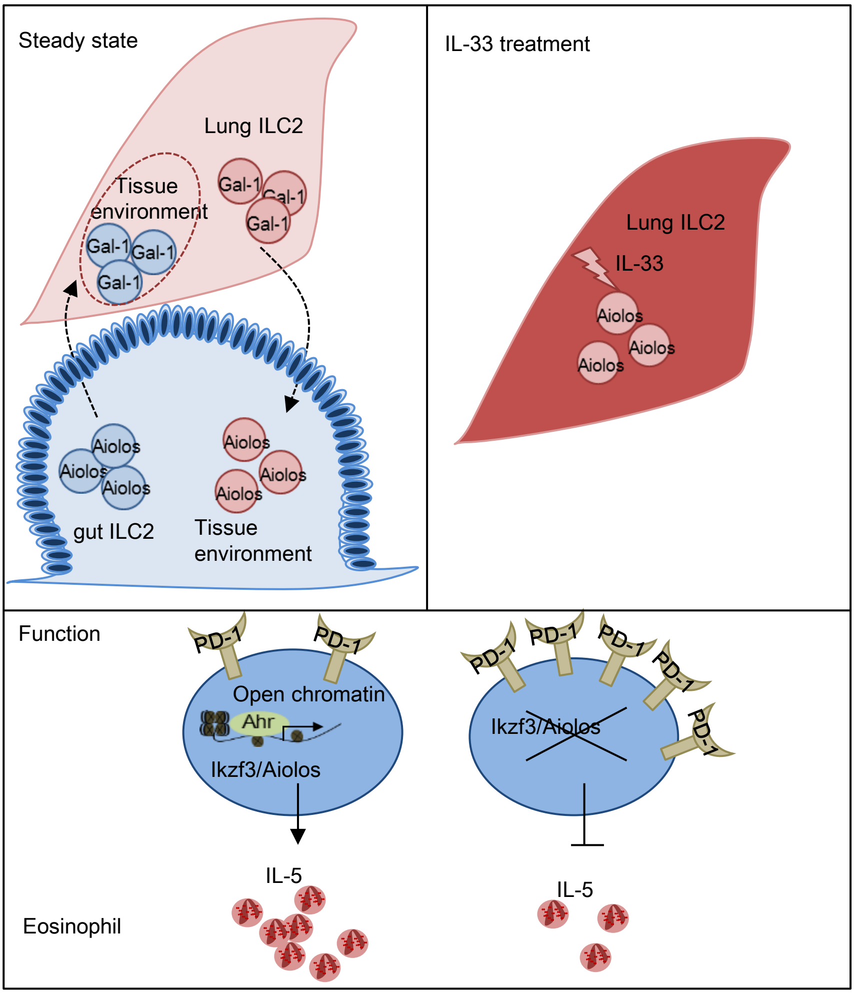

Figure S8 Working model for distinct features of ILC2s shaped by tissue microenvironment

Supplementary Table 1 Genes correlated with *Ikzf3* at single cell level

Genes correlated with *Ikzf3* in mRNA expression at the single cell level was analyzed. *Ikzf3* was labeled as "query gene" and analyzed genes were labeled as "target gene". LI, large intestine.

Genes positively correlated with Ikzf3 expression			
Query gene	Target gene	p value	Pearson correlation coefficient
Ikzf3	*Rora*	0	0.181
Ikzf3	*Tent5a*	0	0.179
Ikzf3	*Il5*	0	0.176
Ikzf3	*Cldnd1*	0	0.167
Ikzf3	*Pdcd1*	0	0.165
Ikzf3	*Sub1*	0	0.165
Ikzf3	*Satb1*	0	0.161
Ikzf3	*Bcl2a1d*	0	0.154
Ikzf3	*Samsn1*	0	0.151
Ikzf3	*Gata3*	0	0.121
Ikzf3	*Bcl2l11*	1.00E-04	0.112
Ikzf3	*Txnip*	0.00296	0.111
Ikzf3	*Areg*	0	0.106
Genes negatively correlated with Ikzf3 expression			
Query gene	Target gene	p value	Pearson correlation coefficient
Ikzf3	*Hspa1a*	6.00E-05	-0.092
Ikzf3	*Fos*	0	-0.09
Ikzf3	*Jun*	0.00144	-0.066

Supplementary Data Legends

Supplementary Data 1 ILC2 tissue specific genes

Analysis of aggregated and down-sampled tissue ILC2 single cell data was performed using Seurat. Genes significantly and differentially expressed over 1.2 fold in indicated tissue ILC2s compared to ILC2s from each of the other tissue through a 1 versus 1 or 1 versus all comparison manner are shown. Log_2(fold change) of the "1 versus all" analysis is shown.

Supplementary Data 2 Fold change of genome accessibility and gene expression of ILC2 tissue specific genes

Analysis of aggregated and down-sampled single cell data from tissue ILC2s was performed using Seurat. ATAC-seq analysis was performed on ILC2s from 5 tissues. Except for the SI and LI ILC2 common genes (see methods), Log_2(Fold change)(Log_2Fc) of mRNA expression (analyzed using a 1 versus all comparison method) and average of Log_2Fc in genome accessibility (ATAC-seq Log2Fc) at different regions of top 35 ILC2 tissue specific genes are shown. BM, bone marrow; LI, large intestine; SI, small intestine.

Supplementary Data 3 Enriched motifs in ILC2 tissue specific OCRs

Significantly enriched motifs, with cutoff p value less than 1×10^{-6}, analyzed by HOMER, in ILC2 tissue specific OCRs are listed. BM, bone marrow; LI, large intestine; SI, small intestine.

Supplementary Data 4 List of antibodies used in this study

Supplementary Data 5 Sequences of primers, shRNAs，guide RNAs and siRNA oligos used in this study
